# Supplementary material for: Swallowed topical steroid therapy for eosinophilic oesophagitis in children: practical, evidence-based guidance by the BSPGHAN Eosinophilic Oesophagitis Working Group
Source: BMJ Paediatr Open. 2024 May 23;8(1):e002467. doi: 10.1136/bmjpo-2023-002467 (PMC11116858; doi:10.1136/bmjpo-2023-002467)
Supplement: Supplementary data [file bmjpo-2023-002467supp001.pdf]

Supplement 1: BSPGHAN EoE WG voting scores for each recommendation

Theme 1 - The BSPGHAN EoE working group recommends that STS are indicated in:

Statement to vote on	Vote received (Score 1-9)
Induction therapy	9,8,9,9,8,9,9,9,9,8,9,9
Maintenance therapy	9,8,9,8,8,9,9,9,9,7,9,9
First line treatment of EoE	9,6,7,8,8,9,9,6,9,8,9,3
Combination therapy with proton pump inhibitors	9,7,9,8,7,7,5,7,9,8,7,5
Second line treatment after unsuccessful responses to dietary therapy and/or proton pump inhibition	9,9,9,9,7,9,9,7,9,8,9,9
Oesophageal stricture treatment in isolation or as an adjunct to oesophageal balloon dilatation	8,6,9,8,6,8,7,7,9,7,1,7

Theme 2 - The BSPGHAN EoE working group recommends that:

Statement to vote on	Vote received (Score 1-9)
The type of STS preparation offered should take into consideration the child's age, palatability, chance of adherence, comorbidities and family support	9,9,9,8,8,9,9,9,9,9,9,9
If a child is assessed to be able to tolerate and coordinate orodispersible budesonide (tablet) preparation and it is locally available, then this preparation should be first choice	8,8,9,7,8,9,9,5,8,7,9,7

Theme 3 - The BSPGHAN EoE working group recommends that:

Statement to vote on	Vote received (Score 1-9)
Age should be used to determine STS dosing, unless a child significantly deviates from their growth centiles (in which case height should be accounted for)	8,8,9,9,8,7,9,9,8,7,9,9

Theme 4 - The BSPGHAN EoE working group recommends that:

Statement to vote on	Vote received (Score 1-9)
Twice daily dosing is considered for induction therapy	8,8,9,8,8,9,7,9,8,8,9,6
For children < 10 years old, a dose of 0.5mg twice per day should be used (1mg/day), for children ≥ 10 years old, a dose of 1mg twice per day should be used (2mg/day)	9,8,8,9,8,8,6,9,9,8,9,9
Induction therapy should usually be given for a minimum of 3 months	9,8,9,8,8,7,9,9,9,8,9,9

Theme 5 - The BSPGHAN EoE working group recommends that:

Statement to vote on	Vote received (Score 1-9)
After histological remission has been achieved, maintenance therapy should be considered for a minimum of 1-2 years	9,8,7,7,8,6,9,9,9,7,9,7
For children < 10 years old, a dose of 0.5mg/day should be used, for children ≥ 10 years old, a dose of 1mg/day should be used	9,8,7,7,8,6,6,9,9,7,9,8

Theme 6 - The BSPGHAN EoE working group recommends that:

Statement to vote on	Vote received (Score 1-9)
After confirmed histological remission and 3-month induction therapy, clinicians should consider halving the STS dose	9,8,8,8,7,7,7,8,8,8,9,9
During maintenance therapy, dose weaning should be considered every 6-12 months	8,8,7,7,7,6,7,8,8,8,4,7
Oral or oesophageal candidiasis does not usually require stopping the STS; antifungal treatment should be added alongside the STS	8,6,7,7,8,6,9,8,8,6,5,5

Theme 7 -The BSPGHAN EoE working group recommends that endoscopy:

Statement to vote on	Vote received (Score 1-9)
Should be repeated during the induction period to ensure histological response to STS and allow weaning of the medication	9,8,8,7,7,9,9,8,8,8,1,9
Should be repeated if there is worsening of symptoms / oesophageal dysfunction	9,9,8,8,9,8,9,8,9,8,9,9
Surveillance should be considered at 1-2 yearly intervals or if considering stopping treatment or following cessation of therapy if clinically indicated	9,8,7,7,9,8,9,8,9,8,9,8
