# Supplementary material for: Swallowed topical steroid therapy for eosinophilic oesophagitis in children: practical, evidence-based guidance by the BSPGHAN Eosinophilic Oesophagitis Working Group
Source: BMJ Paediatr Open. 2024 May 23;8(1):e002467. doi: 10.1136/bmjpo-2023-002467 (PMC11116858; doi:10.1136/bmjpo-2023-002467)
Supplement: Supplementary data [file bmjpo-2023-002467supp002.pdf]

**Appendix 1: How to prepare and use oral viscous budesonide**

In order to dispense the drug in a viscous solution, many different budesonide preparations have been used in local protocols for use in children and practice varies widely[21]. The pharmacokinetic profile of these carrier substances has not been published.

Trials have been completed into a proprietary budesonide oral solution but results have not yet been published and currently no licensed solution is currently available in UK[22].

In the UK there are two strengths of budesonide nebule available that can be mixed into a viscous liquid or slurry; 500microgram in 2ml and 1mg in 2ml. The contents of one nebule is mixed with the adjunct immediately before a dose. Adjuncts commonly used in the UK are given below with currently used dosing.

A summary of international practice states that between 5-15ml of liquid of any sort e.g. apple sauce or honey or any other are used to mix with the budesonide nebuliser solution[21].

| **Adjunct** | **Dose – Weight / volume** | **Dose – Standard household measure / scoops** | **Potential side effects and ingredients** |
|---|---|---|---|
| Splenda Low Calorie Sweetener (also available as Splenda Stevia) | 5g Splenda (sucralose) per 0.5mg (2ml) budesonide nebule | 10 teaspoons (or 3.3 culinary tablespoons of 15ml) or 1 x Nutricia® 50 ml blue scoop.<br>Half the dose of Splenda can be used for a more viscous solution. | Sucralose or Stevia<br>May become contaminated with milk/soya during preparation<br>Potential long-term negative effects described on gut microbiome; cardiovascular risk; diabetes and cancer risk however evidence is limited[23-25] |
| Simple Syrup | 10ml simple syrup per 0.5mg(2ml) budesonide nebule | 2 teaspoons | Sucrose in water |
| Simple Linctus | 10ml Simple Linctus per 0.5mg (2ml) budesonide nebule | 2 teaspoons | Contains citric acid monohydrate, glycerol, aniseed flavour, maltitol liquid (contains sorbitol), sodium benzoate and purified water. |
| Budesonide dispersible tablet | N/A | N/A | Not licenced for under 18 years of age.<br>However evidence of effectiveness in adolescents[26] |

Alternative adjuncts reported in international literature:

| | | | |
|---|---|---|---|
| Apple sauce | 5 g per 0.5mg (2ml) budesonide nebule | 1 teaspoon | Allergic reactions to apple and other ingredients of the sauce[21] |
| Honey | 5 g per 0.5mg (2ml) budesonide nebule | 1 teaspoon | Not to be used in infants under 12 months of age due to botulism risk.<br>Theoretically could contain allergenic substances however allergic reactions to honey are rare[21] |
| Xantham gum | 50mg xanthan gum (0.05g) plus 5ml cooled boiled or sterile water per2ml nebule budesonide | Needs to be measured | Not reported[21] |
