# Supplementary material for: Swallowed topical steroid therapy for eosinophilic oesophagitis in children: practical, evidence-based guidance by the BSPGHAN Eosinophilic Oesophagitis Working Group
Source: BMJ Paediatr Open. 2024 May 23;8(1):e002467. doi: 10.1136/bmjpo-2023-002467 (PMC11116858; doi:10.1136/bmjpo-2023-002467)
Supplement: Author's manuscript [file bmjpo-2023-002467.draft_revisions.pdf]

BMJ Paediatrics Open

BMJ Paediatrics Open is committed to open peer review. As part of this commitment we make the peer review history of every article we publish publicly available.

When an article is published we post the peer reviewers' comments and the authors' responses online. We also post the versions of the paper that were used during peer review. These are the versions that the peer review comments apply to.

The versions of the paper that follow are the versions that were submitted during the peer review process. They are not the versions of record or the final published versions. They should not be cited or distributed as the published version of this manuscript.

BMJ Paediatrics Open is an open access journal and the full, final, typeset and author-corrected version of record of the manuscript is available on our site with no access controls, subscription charges or pay-per-view fees (http://bmjpaedsopen.bmj.com).

If you have any questions on BMJ Paediatrics Open's open peer review process please email info.bmjpo@bmj.com

BMJ Paediatrics Open

Swallowed topical steroid therapy for eosinophilic oesophagitis in children: Practical, evidence-based guidance by the BSPGHAN eosinophilic oesophagitis working group

Journal:	*BMJ Paediatrics Open*
Manuscript ID	bmjpo-2023-002467
Article Type:	Original research
Date Submitted by the Author:	21-Dec-2023
Complete List of Authors:	Chan, Joseph; University Hospital of Wales, Paediatric Gastroenterology<br>Flynn, Diana; Royal Hospital for Children, Paediatric Gastroenterology<br>Gordon, Morris; University of Central Lancashire,<br>Parmar, Raj; Alder Hey Children's Hospital, Paediatric Gastroenterology<br>Moolenschot, Kerryn; St George's Hospital, Clinical Lead Paediatric Dietitian<br>Jackman, Lucy; Great Ormond Street Hospital for Children, Specialist Paediatric Dietitian<br>Gaynor, Ed; Great Ormond Street Hospital for Children, Paediatric Gastroenterology/Mucosal Immunology<br>Epstein, Jenny; Chelsea and Westminster Hospital, Paediatric Gastroenterology<br>Cordell, Amanda; Eosinophilic Family Coalition<br>Kannappan, Hema; University Hospitals Coventry and Warwickshire NHS Trust, General Paediatrics<br>Furman, Mark; Royal Free Hospital, Paediatric Gastroenterology<br>Thompson, Julie; Guts UK<br>Gasparetto, Marco; Norfolk and Norwich University Hospital, Paediatric Gastroenterology<br>Auth, Marcus; Alder Hey Children's NHS Foundation Trust, Gastroenterology, hepatology and nutrition
Keywords:	Gastroenterology

SCHOLARONE™
Manuscripts

I, the Submitting Author has the right to grant and does grant on behalf of all authors of the Work (as defined in the below author licence), an exclusive licence and/or a non-exclusive licence for contributions from authors who are: i) UK Crown employees; ii) where BMJ has agreed a CC-BY licence shall apply, and/or iii) in accordance with the terms applicable for US Federal Government officers or employees acting as part of their official duties; on a worldwide, perpetual, irrevocable, royalty-free basis to BMJ Publishing Group Ltd ("BMJ") its licensees and where the relevant Journal is co-owned by BMJ to the co-owners of the Journal, to publish the Work in this journal and any other BMJ products and to exploit all rights, as set out in our licence.

The Submitting Author accepts and understands that any supply made under these terms is made by BMJ to the Submitting Author unless you are acting as an employee on behalf of your employer or a postgraduate student of an affiliated institution which is paying any applicable article publishing charge ("APC") for Open Access articles. Where the Submitting Author wishes to make the Work available on an Open Access basis (and intends to pay the relevant APC), the terms of reuse of such Open Access shall be governed by a Creative Commons licence – details of these licences and which Creative Commons licence will apply to this Work are set out in our licence referred to above.

Other than as permitted in any relevant BMJ Author's Self Archiving Policies, I confirm this Work has not been accepted for publication elsewhere, is not being considered for publication elsewhere and does not duplicate material already published. I confirm all authors consent to publication of this Work and authorise the granting of this licence.

Confidential: For Review Only

Title

Swallowed topical steroid therapy for eosinophilic oesophagitis in children: Practical, evidence-based guidance by the BSPGHAN eosinophilic oesophagitis working group

Authors' names

Chan J (0000-0002-7281-9619), Flynn DM, Gordon M (0000-0002-1216-5158), Parmar R, Moolenschot K, Jackman L, Gaynor E, Epstein J, Cordell A, Kannappan H, Furman M, Thompson J, Gasparetto M, Auth MKH (0000-0002-9381-6994) on behalf of BSPGHAN Eosinophilic Oesophagitis working group

Address for each author, names and positions

Dr Joseph Chan, Consultant Paediatric Gastroenterologist, University Hospital of Wales, Heath Park Way, Cardiff, CF14 4XW. Email: joe.chan@wales.nhs.uk

Dr Diana M Flynn, Consultant Paediatric Gastroenterologist, Royal Hospital for Children, Glasgow, G51 4TF. Email: diana.flynn@ggc.scot.nhs.uk

Dr Morris Gordon, Consultant Paediatrician with special interest in paediatric gastroenterology, University of Central Lancashire (UCLAN), PR1 2HE. Email: mgordon@uclan.ac.uk

Dr Marco Gasparetto, Consultant Paediatric Gastroenterologist, Norfolk and Norwich University Hospital, University of East Anglia (UEA), NR4 7UY. Email: marco.gasparetto@nhs.net

Dr Marcus KH Auth, Consultant Paediatric Gastroenterologist, Alder Hey Children's Hospital, Liverpool, L12 2AP. Email: Marcus.auth@alderhey.nhs.uk

BSPGHAN Eosinophilic Oesophagitis working group:

Drs MKH Auth, J Chan, DM Flynn, - details as above.

Amanda Cordell, CEO EOS Network, EOS Network - Eosinophilic Diseases Charity. Email: amandacordell@eosnetwork.org

Dr Jenny Epstein, Consultant Paediatric Gastroenterologist. Chelsea and Westminster Hospital, 369 Fulham Road, London, SW10 9NH. Email: jenny.epstein@chelwest.nhs.uk

Dr Mark Furman, Consultant Paediatric Gastroenterologist, Centre for Paediatric Gastroenterology, Royal Free Hospital, Pond Street, London, NW3 2QG. Email: mark.furman@nhs.net

Dr Ed Gaynor, Consultant Paediatric Gastroenterologist, Department of Gastroenterology/Mucosal Immunology, Great Ormond Street Hospital, Great Ormond Street, London, WC1N 3JH. Email: ed.gaynor@nhs.net

Lucy Jackman, Specialist Paediatric Dietitian, Great Ormond Street Hospital, Great Ormond Street, London, WC1N 3JH. Email: lucy.jackman@gosh.nhs.uk

Dr Hema Kannappan, Consultant Paediatrician with special interest in gastroenterology, University Hospital Coventry and Warwickshire, Clifford Bridge Road, Coventry, CV2 2DX. Email: hema.kannappan@uhcw.nhs.uk

Confidential: For Review Only

Kerryn Moolenschot, Clinical Lead Paediatric Dietitian, Paediatric Gastrointestinal Services, St Georges Hospital, Blackshaw Road, London, SW17 0QT. Email: kerryn.moolenschot@stgeorges.nhs.uk

Dr Raj Parmar, Consultant Paediatric Gastroenterologist, Alder Hey Children's Hospital, Liverpool, L12 2AP. Email: raj.parmar@alderhey.nhs.uk

Julie Thompson, Information manager, Guts UK, 2 St Andrews Place, London, NW1 4LB - Guts UK Charity. Email: jthompson@gutscharity.org.uk

Corresponding author

Dr Morris Gordon, Consultant Paediatrician with special interest in paediatric gastroenterology, University of Central Lancashire (UCLAN), PR1 2HE. Email: mgordon@uclan.ac.uk

Confidential: For Review Only

Abstract

Objective: To develop evidence-based guidance for topical steroid use in paediatric Eosinophilic Oesophagitis (pEoE) in the UK, for both induction and maintenance treatment.

Methods: A systematic literature review using Cochrane guidance was carried out by the BSPGHAN Eosinophilic Oesophagitis (EoE) working group (WG) and research leads to determine the evidence base for; preparation, dosing and duration of use of swallowed topical steroid (STS) formulations in EoE. Seven themes relating to pEoE were reviewed by the WG, alongside the Cochrane review this formed the evidence base for consensus recommendations for pEoE in the UK.

Results: 2638 citations were identified and 18 randomised controlled trials were included. Evidence exists for the use of STS for induction and maintenance therapy in EoE, especially regarding histological improvement. Using AGREE criteria, dosing of steroids by age (0.5mg BD <10 years and 1mg BD ≥ 10 years) for induction of at least 3 months was suggested based on evidence and practical consideration. Once histological remission is achieved, maintenance dosing of steroids appears to reduce the frequency and severity of relapse, as such a maintenance weaning regimen is proposed. We provide an overview of practical considerations including dosing regimen and treatment concordance. Oral viscous budesonide (OVB) and, if agreed by local regulatory committees, orodispersible budesonide (budesonide 1mg tablets) were selected for ease of use and with most improvement in histology. A practical 'how to prepare and use' OVB supplement is included (Appendix 1). Side effects identified included candidiasis and adrenal gland suppression. The use of oral systemic steroids in strictures is discussed briefly.

Conclusion: A practical, evidence-based flowchart and guidance recommendations with consensus from the EoE WG and education and research representatives of BSPGHAN was developed with detailed practical considerations for use in the UK.

Confidential: For Review Only

Key Messages

What is already known on this topic

- Therapeutic options for pEoE are limited. STS are the most successful treatment option in adults and children.
- Unlike in adults, currently there is no licensed formulation for a topical steroid in pEoE leaving prescribers with a variety of local dispensing policies.
- These therapies have evidence of benefit for paediatric patients of all ages but exact data on efficacy and safety have been missing in previous guidelines.

What this study adds

- We provide Cochrane evidence-based data on the reported safety and efficacy of topical steroids in children with EoE.
- Where published evidence is sparse or lacking, we provide a national WG consensus for the first time in pEoE.
- This study summarises safety aspects and also pragmatic advice on mode and type of dispensing STS.

How this study might affect research, practice or policy

- These recommendations and flowchart will help to standardise medical care of children with EoE treated with STS both nationally and internationally.
- Implementing a robust scheme for induction and maintenance with mandatory surveillance endoscopies will allow local benchmarking and prospective outcome measures.
- This study will close a gap of clinical research and provide a bridge for future studies on best treatment options and prognosis for children with EoE.

Confidential: For Review Only

Introduction

Eosinophilic oesophagitis (EoE) is a chronic, immune or antigen-mediated oesophageal disease characterised by symptoms of oesophageal dysfunction and eosinophil predominant mucosal inflammation[1] first discovered in the early 1990s[2,3]. The rising prevalence is estimated at 15/100,000 before 2007 and 63/100,000 since 2017[4]. It is more common in males and is associated with atopic diseases. Current incidence estimates range from 5-10/100,000[5] similar to paediatric inflammatory bowel disease in the UK[6]. There has been a need to produce guidance for healthcare professionals and patients but these have mostly been adult focused and not always translatable to paediatric medicine. In the UK, a joint consensus guideline by the British Society of Gastroenterology and the British Society of Paediatric Gastroenterology, Hepatology and Nutrition (BSPGHAN) has attempted to redress this balance and pave the way for a joined up EoE approach[7].

Swallowed topical steroids (STS) have become common practice in recent years for induction and maintenance treatment of EoE, however practical barriers to treatment exist for the paediatric EoE (pEoE) cohort. The diagnosis is normally made in tertiary paediatric gastroenterology settings due to access to endoscopy and paediatric anaesthetists for general anaesthesia, these settings are less accessible for families living outside of major cities. Additionally, the newest orodispersible STS is unlicensed in patients less than 18 years old and is often unavailable in primary and secondary care. Tertiary centres are obliged to continue prescriptions which contributes to increased cost burden to families making long journeys to collect the medication or on hospital pharmacies who fund delivery of the medication to the family. Another barrier for pEoE is obtaining STS that are child-friendly and acceptable to the individual and family. Poor compliance is associated with medications difficult to administer due to product bitterness, time taken to prepare the medication and technical challenges involved in coordinating the swallow in younger age groups.

In the absence of a licensed paediatric formulation, choice of different STS and inequality of access to tertiary healthcare settings, UK practice varies and is not standardised. The BSPGHAN EoE working group (WG) has taken the opportunity to collaborate with BSPGHAN research leads and used Cochrane evidence to address some of the issues faced by children with EoE and provide healthcare professionals with practical, evidence-based guidance and a flowchart to support clinical care in healthcare settings.

Confidential: For Review Only

Methods

The BSPGHAN EoE WG met virtually by teleconference to discuss issues relevant to pEoE and what aspects guidance should focus on. There were four meetings between December 2021 and December 2022. The authors performed a systematic literature review on CENTRAL, MEDLINE, Embase, ClinicalTrials.gov and WHO ICTRP to 3 March 2023, with no prior date limits, using Cochrane guidance[8]. Search criteria were randomised controlled trials (RCTs) comparing any medical intervention or food elimination diet for the treatment of EoE. Dichotomous analysis was undertaken to assess response, either alone or in combination, to any other intervention (including placebo). 2638 citations were identified and 18 RCTs pertaining to steroid use were included. This technical review and GRADE evidence included both adult and paediatric studies and informed the evidence base for the WG recommendations.

The authors and WG reviewed the following 7 themes in paediatric patients (age < 18 years) diagnosed with EoE.

1. What are the indications for STS?
2. Which STS preparations are best and how should they be made up?
3. Should dosing be based on age or height?
4. What dose and duration should induction therapy be?
5. What dose and duration should maintenance therapy be?
6. How and when should STS be reduced or stopped?
7. When should endoscopy be repeated?

Recommendations related to each theme were proposed and the WG voted anonymously on each recommendation. A 9-point scale was used with 9 representing strong agreement and 1 representing strong disagreement. Consensus was reached if more than 80% of eligible voting members voted 6, 7, 8 or 9. All WG members are professionals with expertise in EoE.

Patient and Public Involvement statement

Two members of the WG are affiliated with patient support groups; one is the chief executive officer of the EOSnetwork, an Eosinophilic Disease Charity that supports patients with EoE and their families, the other member is affiliated with GUTS UK!, a charity funding research and supporting people living with gastrointestinal disease. Their involvement has been from the outset of development of this project with contribution and comment on the flowchart, statements and recommendations. They have been integral in considering how the information can be disseminated to the wider EoE community.

Results

All of the recommendations made by the BSPGHAN EoE WG (Table 1) for each pEoE theme reached consensus. Voting breakdowns are provided (Supplement 1).

Confidential: For Review Only

Table 1: Main recommendations from the BSPGHAN EoE WG with voting consensus percentage

Theme	The BSPGHAN EoE WG recommends that:	Voting consensus (%)
1	STS are indicated in:<br>• Induction therapy<br>• Maintenance therapy<br>• First line treatment of EoE<br>• Combination therapy with PPI<br>• Second line treatment after unsuccessful responses to dietary therapy and/or PPI<br>• Oesophageal stricture treatment in isolation or as an adjunct to oesophageal balloon dilatation	 100<br>100<br>92<br>83<br>100<br>92
2	• The type of STS preparation offered should take into consideration the child's age, palatability, chance of adherence, comorbidities and family support<br>• If a child is assessed to be able to tolerate and coordinate orodispersible budesonide (tablet) preparation and it is locally available, then this preparation should be first choice	100<br>92
3	• Age should be used to determine STS dosing unless a child significantly deviates from their growth centiles, in which case height should be accounted for	100
4	• Twice daily dosing is considered for induction therapy<br>• For children < 10 years old, a dose of 0.5mg twice per day should be used (1mg/day), for children ≥ 10 years old, a dose of 1mg twice per day should be used (2mg/day)<br>• Induction therapy should usually be given for a minimum of 3 months	100<br>100<br>100
5	• After histological remission has been achieved, maintenance therapy should be considered for a minimum of 1-2 years<br>• For children < 10 years old, a dose of 0.5mg/day should be used, for children ≥ 10 years old, a dose of 1mg/day should be used	100<br>100
6	• After confirmed histological remission and 3-month induction therapy, clinicians should consider halving the STS dose<br>• During maintenance therapy, dose weaning should be considered every 6-12 months (92%)<br>• Oral or oesophageal candidiasis does not usually require stopping the STS; antifungal treatment should be added alongside the STS	100<br>92<br>83
7	Endoscopy:<br>• Should be repeated during the induction period to ensure histological response to STS and allow weaning of the medication<br>• Should be repeated if there is worsening of symptoms / oesophageal dysfunction<br>• Surveillance should be considered at 1-2 yearly intervals or if considering stopping treatment or following cessation of therapy if clinically indicated	 92<br>100<br>100

Confidential: For Review Only

Theme 1 - What are the indications for STS?

There is strong evidence for the use of STS in EoE. STS have very good remission rates, 71% symptomatically and 59% histologically in children not responding to proton pump inhibitor (PPI) medication[8, 9]. There is emerging evidence showing that combination therapy of STS with PPI may lead to even better outcomes[8]. Currently, there are no data in pEoE showing that the use of STS will prevent oesophageal strictures or that strictures can be treated in isolation with STS. However, in practice, medical treatment is often trialled before therapeutic balloon dilatation. The main evidence supporting steroid use in moderate to severe oesophageal strictures is by using systemic steroids. A multicentre retrospective cohort study found that with systemic steroids 20/20 patients showed clinical improvement, 15/20 became asymptomatic and 19/20 had stricture resolution at endoscopy[10].

Theme 2 - Which STS preparations are best and how should they be made up?

There are three main STS preparations: a) oral viscous budesonide ('budesonide slurry') formed from budesonide respules combined with a viscous binding agent, b) orodispersible budesonide (tablets) which melt on the tongue before being swallowed, c) inhaled fluticasone sprayed into the buccal cavity and swallowed. There are currently no head-to-head trials on different preparations or on the ideal viscous binding agent to use to form the slurry. Expert opinion would advocate using an age appropriate preparation which is generally a budesonide slurry in younger children and orodispersible tablets in older children.

Theme 3 - Should dosing be based on age or height?

Research studies looking at histological remission rates and dosing in children with EoE have used either age ranges (< 10 years or ≥ 10years) or heights (< 1.5metres or ≥ 1.5metres) to define cut offs for medication dosing[11]. Expert opinion would advocate using age for simplicity. If a child deviates from their expected growth centiles then consideration should be given to using height instead of age to determine STS dosing.

Theme 4 - What dose and duration should induction therapy be?

There is a lack of evidence surrounding the duration and dosing regimen of induction therapy, expert consensus would favour a minimum of a 3-month period and twice daily dosing to promote increased contact time between the medication and oesophageal mucosa. In children < 10 years old, a standard total daily dose of 1mg/day in divided doses can be safely used. In children ≥ 10 years old, a standard total daily dose of 2mg/day in divided doses should be used. In select cases doses of up to 2.8mg/day (<10 years old) and 4mg/day (≥ 10 years old) have been used[12]. Clinician judgement should be used to balance up the optimal dosing frequency versus the chance of adherence in particular cases.

Confidential: For Review Only

Theme 5 - What dose and duration should maintenance therapy be?

Once histological remission has been accomplished, use of maintenance dosing of steroids appears to reduce the frequency and severity of relapse [9,13]. There is currently limited evidence regarding how long maintenance therapy should be continued for. Once histological remission has been achieved during the induction phase, maintenance therapy should be considered for a minimum of 1-2 years. Usually, maintenance dosing is started at half the daily induction dose. Consideration should be given to whether once or twice daily dosing is likely to result in the greatest effect and adherence to longer term therapy[14].

Theme 6 - How and when should STS be reduced or stopped?

Histological remission is defined as < 15 eosinophils per high power field or $0.3mm^2$ in any oesophageal biopsy examined (proximal, mid and distal oesophagus). Systemic side effects of topical steroids are considered rare during the long-term treatment of EoE patients in those naïve to STS and without pre-existing adrenal conditions[15]. In a multicentre study from the USA, 5% of children on STS were considered to show signs of adrenal gland insufficiency while on STS for longer than 6 months, however the authors concluded that more data are required to develop a suitable screening tool[12]. Further data from a European multi-centre prospective trial are awaited. Continued monitoring of bone mineral density and adrenal suppression is recommended in children and adolescents[7]. EoE patients often have associated atopic conditions and careful consideration should be given to the total daily steroid dose and steroid burden that these patients receive especially during seasonal exacerbations of their atopic disease. Consideration should be given to maintaining EoE on the lowest practical dose of STS to maintain histological remission.

Theme 7 - When should endoscopy be repeated?

There is no clear paediatric guidance on when an endoscopy should be repeated following diagnosis. In children, histological findings often correlate very poorly with symptoms[9,16]. Endoscopy and biopsy should be used to ensure that STS treatment strategies are effective, enable management changes and investigate worrying symptoms.

Flowchart

By assimilating the key evidence from the literature and Cochrane review (Figure 1), recommendations (Table 1) and expert consensus, a practical easy to use flowchart (Figure 2) was designed to help support healthcare professionals involved in the management of pEoE.

Confidential: For Review Only

Discussion

The incidence, prevalence, recognition and management of EoE has rapidly changed over the past 30 years and will continue to evolve as our understanding and therapeutic options change. It is essential that guidance and recommendations are standardised to ensure patients are not disadvantaged by variations in access to healthcare professionals, specific professional knowledge or medications. Using the latest Cochrane systematic review and through collaboration between a national EoE expert group, BSPGHAN education and research leads and patient support groups, we recommend guidance that addresses common themes in pEoE in the UK. We have produced a quick reference flowchart that can be used at the bedside to help guide professionals involved in the management of pEoE. The flowchart was orally presented at the 2023 BSPGHAN Annual Meeting attended by 130 professionals for wider society member review and discussion. Specific comments were requested using a QR code to ensure stakeholder engagement before wider dissemination. The flowchart was found to be useful by all responders with respondents keen to use it in their practice. Where evidence exists, this has been used to strengthen the recommendations and flowchart and where lacking, expert consensus has been followed.

Efficacy, palatability and adherence

Twice daily dosing theoretically allows for a minimum of a daily anti-inflammatory dose especially if one dose is missed. The WG were advised that for adherence to maintenance therapy once daily dosing is more acceptable[14]. Individual circumstances should be discussed and tailored according to lifestyle and activities. As there are currently no licensed preparations of STS in children, a variety of carrier substances for budesonide STS preparations to coat the oesophagus have been used for administration and palatability (Appendix 1). This is heavily reliant on local expertise and there are no head to head trials to advocate use of one over another. Orodispersible budesonide (tablets) are licenced in adults and are increasingly being used off label. Prospective paediatric studies for STS have been conducted and first results have been submitted for peer-reviewed presentation[17], indicating good prospects for suspensions and tablets, subject to pharmaceutical licencing, for future application in children.

Side effects

Children with EoE have a 70% association with atopic comorbidity, steroids are often prescribed by other physicians (Dermatology, Respiratory, General Paediatrics) which potentially increases the cumulative steroid burden. The largest multicentre study reported that 5% of children showed signs of adrenal gland insufficiency while on STS for EoE for longer than 6 months[16]. In study protocols, baseline cortisol levels, short synacthen testing and follow-up while on maintenance treatment have been applied, however there is controversy of the standardisation and interpretation of test results, we recommend to perform these as clinically indicated and according to local endocrinology advice. Other monitoring should include growth parameters and bone mineral densities. Candida infection is rare and can be treated simultaneously with topical anti-fungal or oral medication[7].

Endoscopy, prognosis, withdrawal and escalation

In the absence of validated clinical activity scores or non-invasive biomarkers, the group agree that endoscopic reassessment with histology should be used after any significant treatment start or modification. As STS are effective in more than 80% of children when taken according to protocols and usually well tolerated, prognosis is very good. Withdrawal is indicated in non-responders, disease progression under therapeutic dosage of STS or clinically relevant adrenal gland insufficiency. We found limited evidence for escalation of treatment with regard to dosage of STS[16,18].

Confidential: For Review Only

Limitations

There is no evidence for the ideal carrier substance used to generate the optimal formulation of STS. There is a lack of studies to compare potential effects of once or twice daily administration of a defined daily dose of STS on efficacy and long-term outcomes. STS have been considered mainly in isolation and combination therapy has not been investigated as they have been discussed elsewhere[8,16,18,19].

Implications for clinicians and policymakers

This project is a starting point for pEoE in the UK and beyond; further work should be considered with other professionals in paediatric practice to develop a collaborative multi-system approach to pEoE care including allergy and transition to adult settings. This would ensure continued evolution of the clinical reference flowchart when better access to medication occurs; merging paediatric and adult practice should be the gold standard to aim for.

Unanswered questions and future research

Increasing the pEoE knowledge base is vital to combat the increasing disease burden. Differentiating if the increased pEoE prevalence is due to earlier recognition or is suggestive of a more severe phenotype may help to risk stratify patients and impact ongoing surveillance and transition decisions. Whilst it is important to research new treatments for EoE and improve access to these in paediatrics, it will be crucial to know whether the stimulation and recruitment of eosinophils into the oesophagus can be prevented. More trials are needed on whether the type, viscosity and volume of the carrier solution affects the efficacy of topical budesonide. Using this guidance as a baseline will allow future research into best practice and prognosis of pEoE.

Conclusion

An evidence-based guideline and flowchart with consensus from the EoE Working Group and education and research representatives of BSPGHAN was developed with detailed practical considerations for use in the UK and beyond.

Ethics Approval Statement

No ethics approval needed

Funding statement

This research received no specific grant from any funding agency in the public, commercial or not-for-profit sectors

Competing Interests statement

Mixed competing interests: All authors have completed the ICMJE uniform disclosure form at http://www.icmje.org/disclosure-of-interest/ and declare: no support from any organisation for the submitted work; MKHA has received research grants and honorariums from BSPGHAN, Guts UK, Dr Falk Pharma, Mead Johnson, Abbott and Nutricia; AC has received grant funding from AZ, Bristol Myers Squibb, AVIR, Allakos, Dr Falk Pharma and Sanofi / Regeneron and advisory panel honorarium was received from AZ, Sanofi / Regeneron, AVIR and Dr Falk Pharma; Guts UK Charity do receive funding for the Falk Awards from Dr Falk Pharma; no other relationships or activities that could appear to have influenced the submitted work.

Confidential: For Review Only

Contributor and guarantor information:

Joseph Chan and Diana Flynn had the idea for this project on behalf of the BSPGHAN EoE Working group. The initial paper search and outline of the article were developed by them. Joseph Chan took the lead on writing the paper. Diana Flynn contributed to the writing of the paper and development of the steroid algorithm.

Morris Gordon performed the detailed Cochrane Review whereby corroborating evidence was obtained and contributed to structure and writing of the paper.

Marco Gasparetto contributed to the writing of the paper, and advice on grading of statements and recommendations.

Marcus KH Auth took senior overview of the development of the paper and algorithm as Chair of the BSPGHAN EoE Working Group, and contributed to writing and editing of the paper.

All members of the BSPGHAN EoE working group contributed to review of the steroid algorithm, voting and corroboration of statements.

Guarantor for the accuracy and clinical governance of the manuscript is Marcus KH Auth as chair of the EoE working group and member of ESPGHAN EGID working group.

The corresponding author attests that all listed authors meet authorship criteria and that no others meeting the criteria have been omitted.

The lead author affirms that the manuscript is an honest, accurate, and transparent account of the study being reported, and that no important aspects of the study have been omitted. Any discrepancies from the study as originally planned have ben explained.

Copyright/licence for publication

The Corresponding Author has the right to grant on behalf of all authors and does grant on behalf of all authors, a worldwide licence to the Publishers and its licensees in perpetuity, in all forms, formats and media (whether known now or created in the future), to i) publish, reproduce, distribute, display and store the Contribution, ii) translate the Contribution into other languages, create adaptations, reprints, include within collections and create summaries, extracts and/or, abstracts of the Contribution, iii) create any other derivative work(s) based on the Contribution, iv) to exploit all subsidiary rights in the Contribution, v) the inclusion of electronic links from the Contribution to third party material where-ever it may be located; and, vi) licence any third party to do any or all of the above.

Confidential: For Review Only

References

1 Furuta GT, Katzka DA. Eosinophilic Esophagitis. *N Engl J Med.* 2015;373:1640-8.

2 Attwood SE, Smyrk TC, Demeester TR, *et al.* Esophageal eosinophilia with dysphagia. A distinct clinicopathologic syndrome. *Dig Dis Sci.* 1993;38:109-16.

3 Straumann A, Spichtin HP, Bernoulli R. [Idiopathic eosinophilic esophagitis: a frequently overlooked disease with typical clinical aspects and discrete endoscopic findings]. *Schweiz Med Wochenschr.* 1994;124:1419-29.

4 Navarro P, Arias Angel, Arias-Gonzalez L, *et al.* Systematic review with meta-analysis: the growing incidence and prevalence of eosinophilic oesophagitis in children and adults in population-based studies. *Aliment Pharmacol Ther.* 2019;49:1116-25.

5 Dellon ES, Hirano I. Epidemiology and natural history of eosinophilic esophagitis. *Gastroenterology.* 2018;154:319-32.

6 Henderson P, Rogers P, Gillett P, et al. The epidemiology and natural history of paediatric inflammatory bowel disease in a UK region: a prospective 14-year study. *Arch Dis Child.* 2012; 97: A53-A54.

7 Dhar A, Haboubi H, Auth M, Attwood S. Eosinophilic oesophagitis: improving diagnosis and therapy – reducing the burden of repeated endoscopy. *Frontline Gastroenterol.* 2022:13(e1):e51-e56. doi: 10.1136/flgastro-2022-102118. eCollection 2022.

8 Franciosi JP, Gordon M, Sinopoulou V, Dellon ES, Gupta SK, Reed CR, et al. Medical treatment of eosinophilic esophagitis. *Cochrane Database Syst Rev* 2023; 2023(7): CD004065. https://www.cochranelibrary.com/cdsr/doi/10.1002/14651858.CD004065.pub4/information

9 Oliva S, Dias JA, Rea F, et al. Characterization of Eosinophilic Esophagitis from the European Pediatric Eosinophilic Esophagitis Registry (pEEr) of ESPGHAN. *J Pediatr Gastr Nut*r. 2022;75(3):325-333.

10 Hoofien A, Rea F, ESPGHAN EGID Working Group et al. Systemic steroids have a role in treating esophageal strictures in paediatric eosinophilic esophagitis. *Dig Liver Dis.* 2021;53(3):324-328.

11 Gupta SK, Hill M, Vitanza JM, Farber RH, Desai NK, et al. Pharmacokinetics of Budesonide Oral Suspension in Children and Adolescents with Eosinophilic Esophagitis. *J Pediatr Gastroenterol Nutr.* 2022 Aug 1;75(2):186-191. doi: 10.1097/MPG.0000000000003482. Epub 2022 Jun 6.PMID: 35666852

12 Gupta SK, Collins MH, Lewis JD, et al. Efficacy and safety of oral budesonide suspension (OBS) in pediatric subjects with eosinophilic esophagitis (EoE): results from the double-blind, placebo-controlled PEER study. *Gastroenterology.* 2011;140:S179.

13 Straumann A, Lucendo AJ, Miehlke S, et al. Budesonide orodispersible tablets maintain remission in a randomized, placebo-controlled trial of patients with eosinophilic esophagitis. *Gastroenterology.* 2020; 159(5): 1672-85 e5.

14 Auth MKH, Chan J, Flynn D, personal communication with A Cordell (Eosnetwork) and J Thompson (Guts UK!), 09-mar-2023.

Confidential: For Review Only

15 Bose P, Kumar S, Nebesio TD, Li C, Hon EC, et al. Adrenal Insufficiency in Children With Eosinophilic Esophagitis Treated With Topical Corticosteroids. *J Pediatr Gastroenterol Nutr.* 2020, 70(3):324-329.

16 Hoofien A, Dias JA, Malamisura M, Rea F, Chong S, et al. Pediatric Eosinophilic Esophagitis: Results of the Retrospective Pediatric Eosinophilic Esophagitis Registry (RetroPEER). *J Pediatr Gastroenterolg Nutr.* 2019 Apr;68(4):552-558.

17 Lucendo AJ, Antunes H, Papadopoulou A, Auth MKH, Dutt S, et al. Budesonide oral suspension is effective and safe in pediatric eosinophilic esophagitis (EoE): results from the induction phase of the randomized, double-blind, placebo-controlled trial PEDEOS-1. Abstracts submitted to UEGW and ESPGHAN 2024, 13-dec-2023.

18 Oliva S, Amil Dias J, Rea F, Malamisura M, Espinheira MC, et al. Characterization of Eosinophilic Esophagitis in a Multinational Prospective Pediatric Cohort: Initial Report from the European Pediatric Eosinophilic Esophagitis Registry (pEEr) of ESPGHAN. *J Pediatr Gastroenterol Nutr.* 2022; 75(3): 325-333.

19 Amil Dias J, Salvatore S, Papadoloulou A, Thomson M, Gutierrez-Junquez C, et al. Updated Guidelines for Diagnosis and Management of Eosinophilic Esophagitis in Pediatrics. Position Paper for Diagnosis and Management by the EGID Working Group of ESPGHAN. *J Pediatric Gastroenterol Nutr 2024, in print.*

Confidential: For Review Only

Figure 1: Key evidence from the Cochrane review. The Cochrane review provides analysis of 52 statements on the use of STS for the endpoints; successful reduction of eosinophils, resolution of symptoms (dichotomous), mean drop in eosinophils and mean reduction in symptom score (continuous)[8]. The key findings are from 41 RCTs with 3253 participants, of which 14 studies compared corticosteroids to placebo for induction of remission in children alone or children and adults.

1.	Corticosteroids lead to a large histological improvement (63% higher), measured dichotomously (RR 11.94, 95% CI 6.56 to 21.75; 12 studies, 978 participants; NNTB = 3; high certainty), and may lead to histological improvement, measured continuously (SMD 1.42, 95% CI 1.02 to 1.82; 5 studies, 449 participants; low certainty).
2.	Corticosteroids may lead to slightly better clinical improvement (20% higher), measured dichotomously (risk ratio (RR) 1.74, 95% CI 1.08 to 2.80; 6 studies, 583 participants; number needed to treat for an additional beneficial outcome (NNTB) = 4; low certainty), and may lead to slightly better clinical improvement, measured continuously (standard mean difference (SMD) 0.51, 95% CI 0.17 to 0.85; 5 studies, 475 participants; low certainty).
3.	Corticosteroids may lead to little to no endoscopic improvement, measured dichotomously (RR 2.60, 95% CI 0.82 to 8.19; 5 studies, 596 participants; low certainty), and may lead to endoscopic improvement, measured continuously (SMD 1.33, 95% CI 0.59 to 2.08; 5 studies, 596 participants; low certainty).
4.	Corticosteroids may lead to slightly fewer withdrawals due to adverse events (RR 0.64, 95% CI 0.43 to 0.96; 14 studies, 1032 participants; low certainty) and patients probably experience a similar number of both serious side effects and side effects in total, compared to placebo. There may be no difference between corticosteroids and placebo in the improvement of quality of life.

Confidential: For Review Only

Figure 2: Swallowed topical budesonide flowchart for pEoE (Quick clinical reference guide)

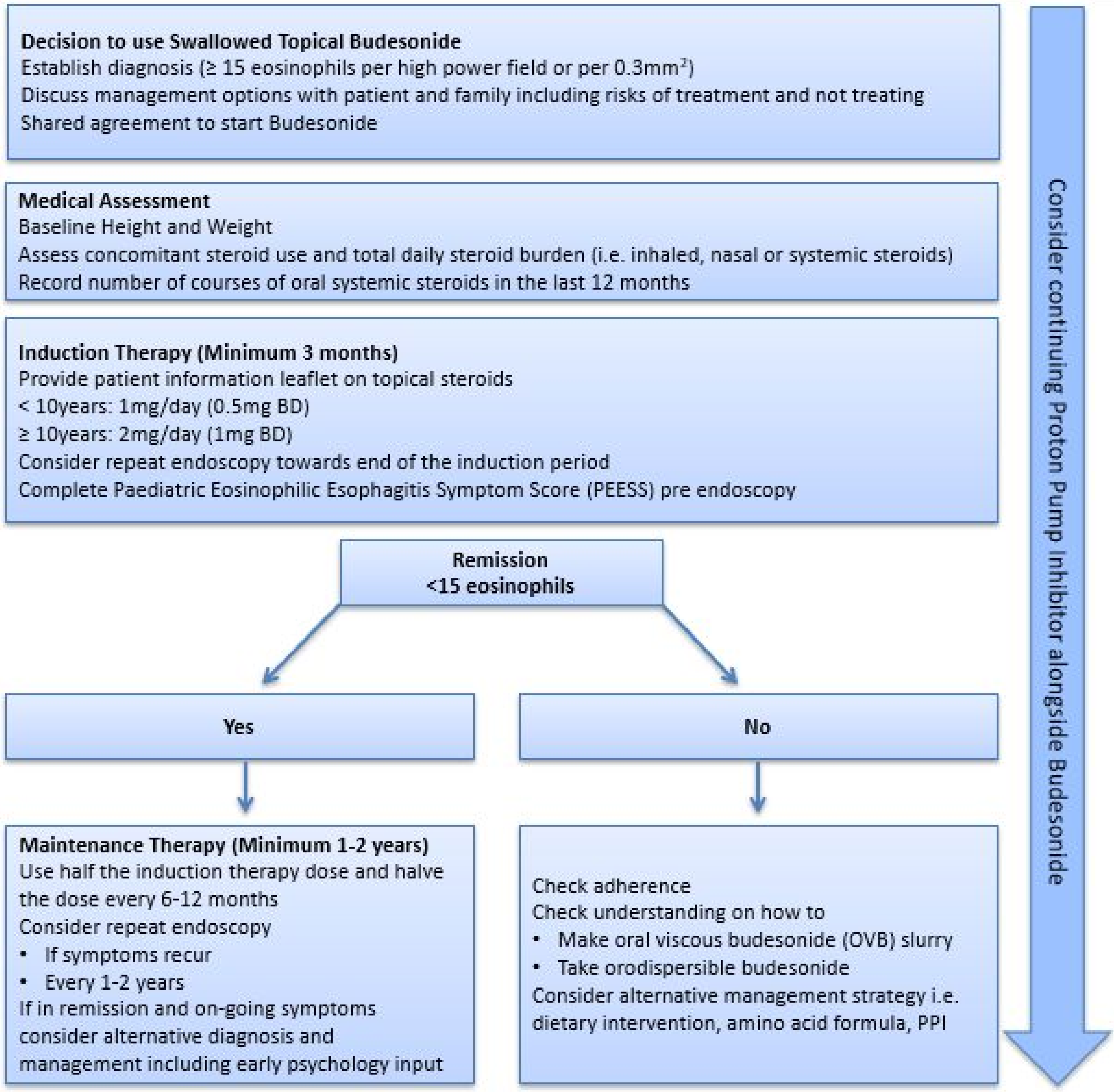

Example Budesonide weaning	<10 years	≥10 years
Induction (0-3 months)	1mg/day	2mg/day
Maintenance (3-15 months)	0.5mg/day	1mg/day
Maintenance (15-27 months)	0.25mg/day	0.5mg/day
Maintenance (27+ months)	0.125mg/day	0.25mg/day
Long term remission	Consider stopping	Consider stopping

Supplement 1: BSPGHAN EoE WG voting scores for each recommendation

Theme 1 - The BSPGHAN EoE working group recommends that STS are indicated in:

Statement to vote on	Vote received (Score 1-9)
Induction therapy	9,8,9,9,8,9,9,9,9,8,9,9
Maintenance therapy	9,8,9,8,8,9,9,9,9,7,9,9
First line treatment of EoE	9,6,7,8,8,9,9,6,9,8,9,3
Combination therapy with proton pump inhibitors	9,7,9,8,7,7,5,7,9,8,7,5
Second line treatment after unsuccessful responses to dietary therapy and/or proton pump inhibition	9,9,9,9,7,9,9,7,9,8,9,9
Oesophageal stricture treatment in isolation or as an adjunct to oesophageal balloon dilatation	8,6,9,8,6,8,7,7,9,7,1,7

Theme 2 - The BSPGHAN EoE working group recommends that:

Statement to vote on	Vote received (Score 1-9)
The type of STS preparation offered should take into consideration the child's age, palatability, chance of adherence, comorbidities and family support	9,9,9,8,8,9,9,9,9,9,9,9
If a child is assessed to be able to tolerate and coordinate orodispersible budesonide (tablet) preparation and it is locally available, then this preparation should be first choice	8,8,9,7,8,9,9,5,8,7,9,7

Theme 3 - The BSPGHAN EoE working group recommends that:

Statement to vote on	Vote received (Score 1-9)
Age should be used to determine STS dosing, unless a child significantly deviates from their growth centiles (in which case height should be accounted for)	8,8,9,9,8,7,9,9,8,7,9,9

Theme 4 - The BSPGHAN EoE working group recommends that:

Statement to vote on	Vote received (Score 1-9)
Twice daily dosing is considered for induction therapy	8,8,9,8,8,9,7,9,8,8,9,6
For children < 10 years old, a dose of 0.5mg twice per day should be used (1mg/day), for children ≥ 10 years old, a dose of 1mg twice per day should be used (2mg/day)	9,8,8,9,8,8,6,9,9,8,9,9
Induction therapy should usually be given for a minimum of 3 months	9,8,9,8,8,7,9,9,9,8,9,9

Confidential: For Review Only

Theme 5 - The BSPGHAN EoE working group recommends that:

Statement to vote on	Vote received (Score 1-9)
After histological remission has been achieved, maintenance therapy should be considered for a minimum of 1-2 years	9,8,7,7,8,6,9,9,9,7,9,7
For children < 10 years old, a dose of 0.5mg/day should be used, for children ≥ 10 years old, a dose of 1mg/day should be used	9,8,7,7,8,6,6,9,9,7,9,8

Theme 6 - The BSPGHAN EoE working group recommends that:

Statement to vote on	Vote received (Score 1-9)
After confirmed histological remission and 3-month induction therapy, clinicians should consider halving the STS dose	9,8,8,8,7,7,7,8,8,8,9,9
During maintenance therapy, dose weaning should be considered every 6-12 months	8,8,7,7,7,6,7,8,8,8,4,7
Oral or oesophageal candidiasis does not usually require stopping the STS; antifungal treatment should be added alongside the STS	8,6,7,7,8,6,9,8,8,6,5,5

Theme 7 -The BSPGHAN EoE working group recommends that endoscopy:

Statement to vote on	Vote received (Score 1-9)
Should be repeated during the induction period to ensure histological response to STS and allow weaning of the medication	9,8,8,7,7,9,9,8,8,8,1,9
Should be repeated if there is worsening of symptoms / oesophageal dysfunction	9,9,8,8,9,8,9,8,9,8,9,9
Surveillance should be considered at 1-2 yearly intervals or if considering stopping treatment or following cessation of therapy if clinically indicated	9,8,7,7,9,8,9,8,9,8,9,8

Confidential: For Review Only

Appendix 1: How to prepare and use oral viscous budesonide

In order to dispense the drug in a viscous solution, many different budesonide preparations have been used in local protocols for use in children and practice varies widely[1]. The pharmacokinetic profile of these carrier substances has not been published.

Trials have been completed into a proprietary budesonide oral solution but results have not yet been published and currently no licensed solution is currently available in UK[2].

In the UK there are two strengths of budesonide nebule available that can be mixed into a viscous liquid or slurry; 500microgram in 2ml and 1mg in 2ml. The contents of one nebule is mixed with the adjunct immediately before a dose. Adjuncts commonly used in the UK are given below with currently used dosing.

A summary of international practice states that between 5-15ml of liquid of any sort eg apple sauce or honey or any other are used to mix with the budesonide nebuliser solution[1].

**Adjunct**	**Dose – Weight / volume**	**Dose – Standard household measure / scoops**	**Potential side effects and ingredients**
Splenda Low Calorie Sweetener (also available as Splenda Stevia)	5g Splenda (sucralose) per 0.5mg (2ml) budesonide nebule	10 teaspoons (or 3.3 culinary tablespoons of 15ml) or 1 x Nutricia® 50 ml blue scoop.<br><br>Half the dose of Splenda can be used for a more viscous solution.	Sucralose or Stevia<br><br>May become contaminated with milk/soya during preparation<br><br>Potential long-term negative effects described on gut microbiome; cardiovascular risk; diabetes and cancer risk however evidence is limited[4,5,6]
Simple Syrup	10ml simple syrup per 0.5mg(2ml) budesonide nebule	2 teaspoons	Sucrose in water
Simple Linctus	10ml Simple Linctus per 0.5mg (2ml) budesonide nebule	2 teaspoons	Contains citric acid monohydrate, glycerol, aniseed flavour, maltitol liquid (contains sorbitol), sodium benzoate and purified water.
Budesonide dispersible tablet	N/A	N/A	Not licenced for under 18 years of age.<br><br>However evidence of effectiveness in adolescents[7]

Confidential: For Review Only

Alternative adjuncts reported in international literature:

Apple sauce	5 g per 0.5mg (2ml) budesonide nebule	1 teaspoon	Allergic reactions to apple and other ingredients of the sauce[1]
Honey	5 g per 0.5mg (2ml) budesonide nebule	1 teaspoon	Not to be used in infants under 12 months of age due to botulism risk.<br>Theoretically could contain allergenic substances however allergic reactions to honey are rare[1]
Xantham gum	50mg xanthan gum (0.05g) plus 5ml cooled boiled or sterile water per2ml nebule budesonide	Needs to be measured	Not reported[1]

Confidential: For Re

References for appendix 1

1. Syverson EP, Tobin M, Patton T, Franciosi JP, Gupta SK, et al. Variability in Swallowed Topical Corticosteroid Practice Patterns for Treatment of Pediatric Eosinophilic Esophagitis. J Pediatr Gastroenterol Nutr 2023 Aug 1;77(2):256-259. doi: 10.1097/MPG.0000000000003834. Epub 2023 May 19.
2. Mukkada VA, Gupta SK, Gold BD, Dellon ES, Collins MH, et al. Pooled Phase 2 and 3 Efficacy and Safety Data on Budesonide Oral Suspension in Adolescents with Eosinophilic Esophagitis. J Pediatr Gastroenterol Nutr. 2023 Sep 18. doi: 10.1097/MPG.0000000000003948. Online ahead of print.PMID: 37718471.
3. Jackman L, Kite K, Aris K, Goh L, Gaynor E. OC17 Oral viscous budesonide (OVB) technique: a patient adherence improvement project. Frontline Gastroenterology Jul 2023, 14 (Suppl 1) A12-A13; DOI: 10.1136/flgastro-2023-bspghan.17.
4. Debras C, Chazelas E, Srour B, Druesne-Pecollo N, Esseddik Y, et al. Artificial sweeteners and cancer risk: Results from the NutriNet-Santé population-based cohort stud. PLoS Med. 2022 Mar; 19(3): e1003950. Published online 2022 Mar 24. doi: 10.1371/journal.pmed.1003950.. Debras et al.
5. Debras C, Deschasaux-Tanguy M, Chazelas E, Sellem L, Druesne-Pecollo N, Esseddik Y Artificial Sweeteners and Risk of Type 2 Diabetes in the Prospective NutriNet-Santé Cohort.Diabetes Care. 2023 Sep 1;46(9):1681-1690. doi: 10.2337/dc23-0206
6. Debras C, Chazelas E, Sellem L, Porcher R, Druesne-Pecollo N. et al. Artificial sweeteners and risk of cardiovascular diseases: results from the prospective NutriNet-Santé cohort. BMJ. 2022 Sep 7;378:e071204. doi: 10.1136/bmj-2022-071204.

7. Mukkada VA, Gupta SK, Gold BD, Dellon ES, Collins MH, Katzka DA, Falk GW, Zhang W, Boules M, Hirano I, Desai N. Pooled Phse 2 nd 3 Efficacy and Safety Data on Budesonide Oral Suspension in Adolescnets with Eosinophilic Oesophagitis. J Pediatr Gastroenterol Nutr. 2023 Sep 18;77(6):760-768. doi: 10.1097/MPG.0000000000003948. Online ahead of print.

Confidential: For Review Only

BMJ Paediatrics Open

Swallowed topical steroid therapy for eosinophilic oesophagitis in children: Practical, evidence-based guidance by the BSPGHAN eosinophilic oesophagitis working group

Journal:	*BMJ Paediatrics Open*
Manuscript ID	bmjpo-2023-002467.R1
Article Type:	Original research
Date Submitted by the Author:	29-Mar-2024

Complete List of Authors:	Chan, Joseph; University Hospital of Wales, Paediatric Gastroenterology<br>Flynn, Diana; Royal Hospital for Children, Paediatric Gastroenterology<br>Gordon, Morris; University of Central Lancashire,<br>Parmar, Raj; Alder Hey Children's Hospital, Paediatric Gastroenterology<br>Moolenschot, Kerryn; St George's Hospital, Clinical Lead Paediatric Dietitian<br>Jackman, Lucy; Great Ormond Street Hospital for Children, Specialist Paediatric Dietitian<br>Gaynor, Ed; Great Ormond Street Hospital for Children, Paediatric Gastroenterology/Mucosal Immunology<br>Epstein, Jenny; Chelsea and Westminster Hospital, Paediatric Gastroenterology<br>Cordell, Amanda; Eosinophilic Family Coalition<br>Kannappan, Hema; University Hospitals Coventry and Warwickshire NHS Trust, General Paediatrics<br>Furman, Mark; Royal Free Hospital, Paediatric Gastroenterology<br>Thompson, Julie; Guts UK<br>Gasparetto, Marco; Norfolk and Norwich University Hospital, Paediatric Gastroenterology<br>Auth, Marcus; Alder Hey Children's NHS Foundation Trust, Gastroenterology, hepatology and nutrition

Keywords:	Gastroenterology, Therapeutics, Child Health

SCHOLARONE™
Manuscripts

I, the Submitting Author has the right to grant and does grant on behalf of all authors of the Work (as defined in the below author licence), an exclusive licence and/or a non-exclusive licence for contributions from authors who are: i) UK Crown employees; ii) where BMJ has agreed a CC-BY licence shall apply, and/or iii) in accordance with the terms applicable for US Federal Government officers or employees acting as part of their official duties; on a worldwide, perpetual, irrevocable, royalty-free basis to BMJ Publishing Group Ltd ("BMJ") its licensees and where the relevant Journal is co-owned by BMJ to the co-owners of the Journal, to publish the Work in this journal and any other BMJ products and to exploit all rights, as set out in our licence.

The Submitting Author accepts and understands that any supply made under these terms is made by BMJ to the Submitting Author unless you are acting as an employee on behalf of your employer or a postgraduate student of an affiliated institution which is paying any applicable article publishing charge ("APC") for Open Access articles. Where the Submitting Author wishes to make the Work available on an Open Access basis (and intends to pay the relevant APC), the terms of reuse of such Open Access shall be governed by a Creative Commons licence – details of these licences and which Creative Commons licence will apply to this Work are set out in our licence referred to above.

Other than as permitted in any relevant BMJ Author's Self Archiving Policies, I confirm this Work has not been accepted for publication elsewhere, is not being considered for publication elsewhere and does not duplicate material already published. I confirm all authors consent to publication of this Work and authorise the granting of this licence.

Confidential: For Review Only

Figure 1: Key evidence from the Cochrane review. The Cochrane review provides analysis of 52 statements on the use of STS for the endpoints; successful reduction of eosinophils, resolution of symptoms (dichotomous), mean drop in eosinophils and mean reduction in symptom score (continuous)[8]. The key findings are from 41 RCTs with 3253 participants, of which 14 studies compared corticosteroids to placebo for induction of remission in children alone or children and adults.

1.	Corticosteroids lead to a large histological improvement (63% higher), measured dichotomously (RR 11.94, 95% CI 6.56 to 21.75; 12 studies, 978 participants; NNTB = 3; high certainty), and may lead to histological improvement, measured continuously (SMD 1.42, 95% CI 1.02 to 1.82; 5 studies, 449 participants; low certainty).
2.	Corticosteroids may lead to slightly better clinical improvement (20% higher), measured dichotomously (risk ratio (RR) 1.74, 95% CI 1.08 to 2.80; 6 studies, 583 participants; number needed to treat for an additional beneficial outcome (NNTB) = 4; low certainty), and may lead to slightly better clinical improvement, measured continuously (standard mean difference (SMD) 0.51, 95% CI 0.17 to 0.85; 5 studies, 475 participants; low certainty).
3.	Corticosteroids may lead to little to no endoscopic improvement, measured dichotomously (RR 2.60, 95% CI 0.82 to 8.19; 5 studies, 596 participants; low certainty), and may lead to endoscopic improvement, measured continuously (SMD 1.33, 95% CI 0.59 to 2.08; 5 studies, 596 participants; low certainty).
4.	Corticosteroids may lead to slightly fewer withdrawals due to adverse events (RR 0.64, 95% CI 0.43 to 0.96; 14 studies, 1032 participants; low certainty) and patients probably experience a similar number of both serious side effects and side effects in total, compared to placebo. There may be no difference between corticosteroids and placebo in the improvement of quality of life.

Confidential: For Review Only

Figure 2: Swallowed Topical Budesonide Flowchart (Quick Clinical Reference Guide)

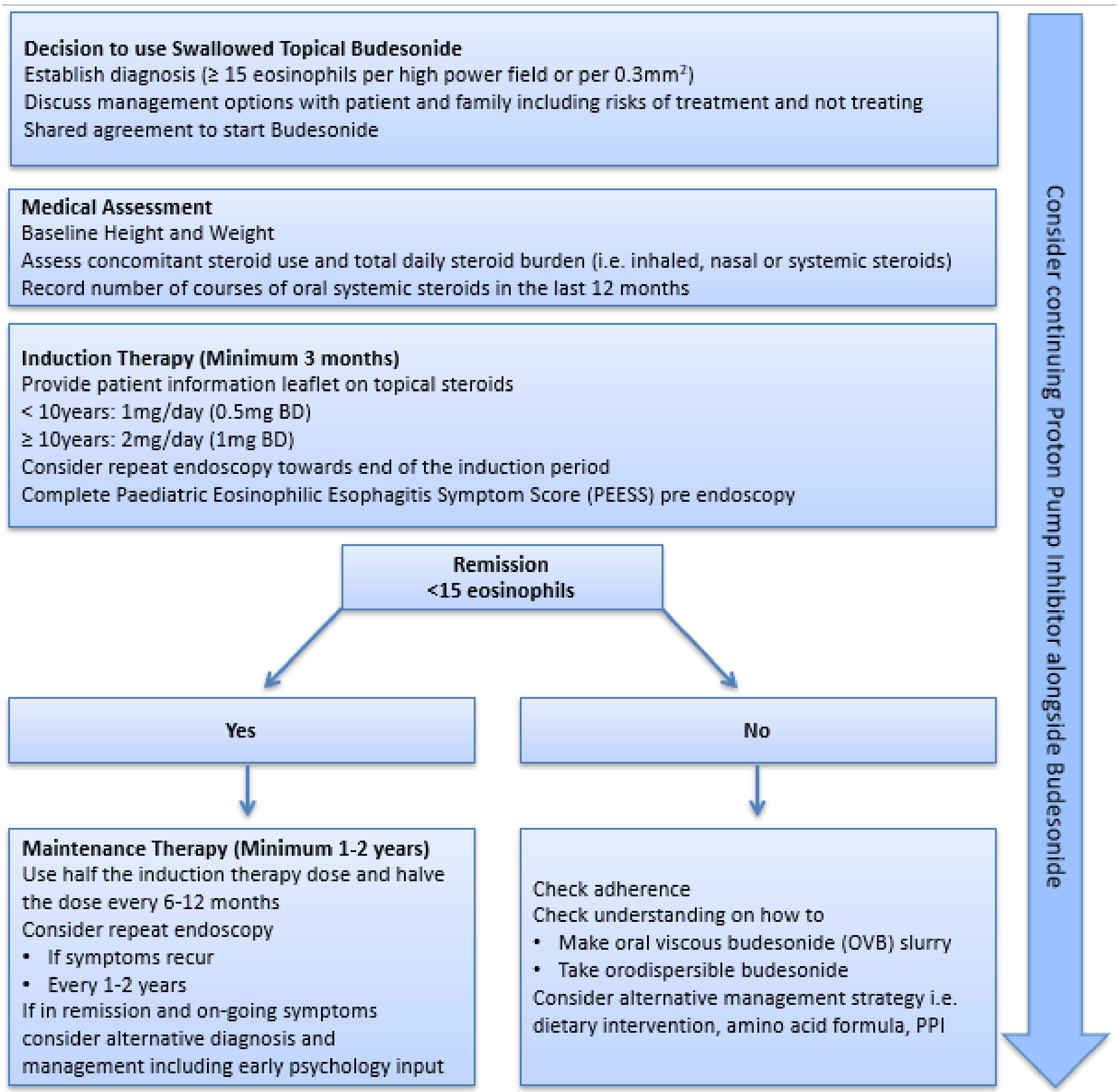

Example Budesonide weaning	<10 years	≥10 years
Induction (0-3 months)	1mg/day	2mg/day
Maintenance (3-15 months)	0.5mg/day	1mg/day
Maintenance (15-27 months)	0.25mg/day	0.5mg/day
Maintenance (27+ months)	0.125mg/day	0.25mg/day
Long term remission	Consider stopping	Consider stopping

Supplement 1: BSPGHAN EoE WG voting scores for each recommendation

Theme 1 - The BSPGHAN EoE working group recommends that STS are indicated in:

Statement to vote on	Vote received (Score 1-9)
Induction therapy	9,8,9,9,8,9,9,9,9,8,9,9
Maintenance therapy	9,8,9,8,8,9,9,9,9,7,9,9
First line treatment of EoE	9,6,7,8,8,9,9,6,9,8,9,3
Combination therapy with proton pump inhibitors	9,7,9,8,7,7,5,7,9,8,7,5
Second line treatment after unsuccessful responses to dietary therapy and/or proton pump inhibition	9,9,9,9,7,9,9,7,9,8,9,9
Oesophageal stricture treatment in isolation or as an adjunct to oesophageal balloon dilatation	8,6,9,8,6,8,7,7,9,7,1,7

Theme 2 - The BSPGHAN EoE working group recommends that:

Statement to vote on	Vote received (Score 1-9)
The type of STS preparation offered should take into consideration the child's age, palatability, chance of adherence, comorbidities and family support	9,9,9,8,8,9,9,9,9,9,9,9
If a child is assessed to be able to tolerate and coordinate orodispersible budesonide (tablet) preparation and it is locally available, then this preparation should be first choice	8,8,9,7,8,9,9,5,8,7,9,7

Theme 3 - The BSPGHAN EoE working group recommends that:

Statement to vote on	Vote received (Score 1-9)
Age should be used to determine STS dosing, unless a child significantly deviates from their growth centiles (in which case height should be accounted for)	8,8,9,9,8,7,9,9,8,7,9,9

Theme 4 - The BSPGHAN EoE working group recommends that:

Statement to vote on	Vote received (Score 1-9)
Twice daily dosing is considered for induction therapy	8,8,9,8,8,9,7,9,8,8,9,6
For children < 10 years old, a dose of 0.5mg twice per day should be used (1mg/day), for children ≥ 10 years old, a dose of 1mg twice per day should be used (2mg/day)	9,8,8,9,8,8,6,9,9,8,9,9
Induction therapy should usually be given for a minimum of 3 months	9,8,9,8,8,7,9,9,9,8,9,9

Theme 5 - The BSPGHAN EoE working group recommends that:

Statement to vote on	Vote received (Score 1-9)
After histological remission has been achieved, maintenance therapy should be considered for a minimum of 1-2 years	9,8,7,7,8,6,9,9,9,7,9,7
For children < 10 years old, a dose of 0.5mg/day should be used, for children ≥ 10 years old, a dose of 1mg/day should be used	9,8,7,7,8,6,6,9,9,7,9,8

Theme 6 - The BSPGHAN EoE working group recommends that:

Statement to vote on	Vote received (Score 1-9)
After confirmed histological remission and 3-month induction therapy, clinicians should consider halving the STS dose	9,8,8,8,7,7,7,8,8,8,9,9
During maintenance therapy, dose weaning should be considered every 6-12 months	8,8,7,7,7,6,7,8,8,8,4,7
Oral or oesophageal candidiasis does not usually require stopping the STS; antifungal treatment should be added alongside the STS	8,6,7,7,8,6,9,8,8,6,5,5

Theme 7 -The BSPGHAN EoE working group recommends that endoscopy:

Statement to vote on	Vote received (Score 1-9)
Should be repeated during the induction period to ensure histological response to STS and allow weaning of the medication	9,8,8,7,7,9,9,8,8,8,1,9
Should be repeated if there is worsening of symptoms / oesophageal dysfunction	9,9,8,8,9,8,9,8,9,8,9,9
Surveillance should be considered at 1-2 yearly intervals or if considering stopping treatment or following cessation of therapy if clinically indicated	9,8,7,7,9,8,9,8,9,8,9,8

Appendix 1: How to prepare and use oral viscous budesonide

In order to dispense the drug in a viscous solution, many different budesonide preparations have been used in local protocols for use in children and practice varies widely[21]. The pharmacokinetic profile of these carrier substances has not been published.

Trials have been completed into a proprietary budesonide oral solution but results have not yet been published and currently no licensed solution is currently available in UK[22].

In the UK there are two strengths of budesonide nebule available that can be mixed into a viscous liquid or slurry; 500microgram in 2ml and 1mg in 2ml. The contents of one nebule is mixed with the adjunct immediately before a dose. Adjuncts commonly used in the UK are given below with currently used dosing.

A summary of international practice states that between 5-15ml of liquid of any sort e.g. apple sauce or honey or any other are used to mix with the budesonide nebuliser solution[21].

**Adjunct**	**Dose – Weight / volume**	**Dose – Standard household measure / scoops**	**Potential side effects and ingredients**
Splenda Low Calorie Sweetener (also available as Splenda Stevia)	5g Splenda (sucralose) per 0.5mg (2ml) budesonide nebule	10 teaspoons (or 3.3 culinary tablespoons of 15ml) or 1 x Nutricia® 50 ml blue scoop.<br>Half the dose of Splenda can be used for a more viscous solution.	Sucralose or Stevia<br>May become contaminated with milk/soya during preparation<br>Potential long-term negative effects described on gut microbiome; cardiovascular risk; diabetes and cancer risk however evidence is limited[23-25]
Simple Syrup	10ml simple syrup per 0.5mg(2ml) budesonide nebule	2 teaspoons	Sucrose in water
Simple Linctus	10ml Simple Linctus per 0.5mg (2ml) budesonide nebule	2 teaspoons	Contains citric acid monohydrate, glycerol, aniseed flavour, maltitol liquid (contains sorbitol), sodium benzoate and purified water.
Budesonide dispersible tablet	N/A	N/A	Not licenced for under 18 years of age.<br>However evidence of effectiveness in adolescents[26]

Confidential: For Review Only

Alternative adjuncts reported in international literature:

Apple sauce	5 g per 0.5mg (2ml) budesonide nebule	1 teaspoon	Allergic reactions to apple and other ingredients of the sauce[21]
Honey	5 g per 0.5mg (2ml) budesonide nebule	1 teaspoon	Not to be used in infants under 12 months of age due to botulism risk.<br><br>Theoretically could contain allergenic substances however allergic reactions to honey are rare[21]
Xantham gum	50mg xanthan gum (0.05g) plus 5ml cooled boiled or sterile water per2ml nebule budesonide	Needs to be measured	Not reported[21]

Confidential: For Review Only

Title

Swallowed topical steroid therapy for eosinophilic oesophagitis in children: Practical, evidence-based guidance by the BSPGHAN eosinophilic oesophagitis working group

Authors' names

Chan J (0000-0002-7281-9619), Flynn DM, Gordon M (0000-0002-1216-5158), Parmar R, Moolenschot K, Jackman L, Gaynor E, Epstein J, Cordell A, Kannappan H, Furman M, Thompson J, Gasparetto M, Auth MKH (0000-0002-9381-6994)

Address for each author, names and positions

Dr Joseph Chan, Consultant Paediatric Gastroenterologist, University Hospital of Wales, Heath Park Way, Cardiff, CF14 4XW. Email: joe.chan@wales.nhs.uk

Dr Diana M Flynn, Consultant Paediatric Gastroenterologist, Royal Hospital for Children, Glasgow, G51 4TF. Email: diana.flynn@ggc.scot.nhs.uk

Dr Morris Gordon, Consultant Paediatrician with special interest in paediatric gastroenterology, University of Central Lancashire (UCLAN), PR1 2HE. Email: mgordon@uclan.ac.uk

Dr Raj Parmar, Consultant Paediatric Gastroenterologist, Alder Hey Children's Hospital, Liverpool, L12 2AP. Email: raj.parmar@alderhey.nhs.uk

Kerryn Moolenschot, Lead Paediatric Dietitian, Paediatric Gastrointestinal Services, St Georges Hospital, Blackshaw Road, London, SW17 0QT. Email: kerryn.moolenschot@stgeorges.nhs.uk

Lucy Jackman, Specialist Paediatric Dietitian, Great Ormond Street Hospital, Great Ormond Street, London, WC1N 3JH. Email: lucy.jackman@gosh.nhs.uk

Dr Ed Gaynor, Consultant Paediatric Gastroenterologist, Department of Gastroenterology/Mucosal Immunology, Great Ormond Street Hospital, Great Ormond Street, London, WC1N 3JH. Email: ed.gaynor@nhs.net

Dr Jenny Epstein, Consultant Paediatric Gastroenterologist. Chelsea and Westminster Hospital, 369 Fulham Road, London, SW10 9NH. Email: jenny.epstein@chelwest.nhs.uk

Amanda Cordell, CEO EOS Network, EOS Network - Eosinophilic Diseases Charity. Email: amandacordell@eosnetwork.org

Dr Hema Kannappan, Consultant Paediatrician with special interest in gastroenterology, University Hospital Coventry and Warwickshire, Clifford Bridge Road, Coventry, CV2 2DX. Email: hema.kannappan@uhcw.nhs.uk

Dr Mark Furman, Consultant Paediatric Gastroenterologist, Centre for Paediatric Gastroenterology, Royal Free Hospital, Pond Street, London, NW3 2QG. Email: mark.furman@nhs.net

Julie Thompson, Information manager, Guts UK, 2 St Andrews Place, London, NW1 4LB - Guts UK Charity. Email: jthompson@gutscharity.org.uk

Dr Marco Gasparetto, Consultant Paediatric Gastroenterologist, Norfolk and Norwich University Hospital, University of East Anglia (UEA), NR4 7UY. Email: marco.gasparetto@nhs.net

Confidential: For Review Only

Dr Marcus KH Auth, Consultant Paediatric Gastroenterologist, Alder Hey Children's NHS Foundation Trust and University of Liverpool, Liverpool, L12 2AP. Email: Marcus.auth@alderhey.nhs.uk

Corresponding author

Dr Morris Gordon, Consultant Paediatrician with special interest in paediatric gastroenterology, University of Central Lancashire (UCLAN), PR1 2HE. Email: mgordon@uclan.ac.uk

Confidential: For Review Only

Abstract

Objective: To develop evidence-based guidance for topical steroid use in paediatric eosinophilic oesophagitis (pEoE) in the UK, for both induction and maintenance treatment.

Methods: A systematic literature review using Cochrane guidance was carried out by the British Society for Paediatric Gastroenterology, Hepatology and Nutrition (BSPGHAN) Eosinophilic Oesophagitis (EoE) working group (WG) and research leads to determine the evidence base for; preparation, dosing and duration of use of swallowed topical steroid (STS) formulations in EoE. Seven themes relating to pEoE were reviewed by the WG, alongside the Cochrane review this formed the evidence base for consensus recommendations for pEoE in the UK. We provide an overview of practical considerations including treatment regimen and dosing. Oral viscous budesonide (OVB) and, if agreed by local regulatory committees, orodispersible budesonide (budesonide 1mg tablets) were selected for ease of use and with most improvement in histology. A practical 'how to prepare and use' OVB appendix is included (Appendix 1). Side effects identified included candidiasis and adrenal gland suppression. The use of oral systemic steroids in strictures is discussed briefly.

Results: 2638 citations were identified and 18 randomised controlled trials were included. Evidence exists for the use of STS for induction and maintenance therapy in EoE, especially regarding histological improvement. Using AGREE criteria, dosing of steroids by age (0.5mg BD <10 years and 1 mg BD ≥ 10 years) for induction of at least 3 months was suggested based on evidence and practical consideration. Once histological remission is achieved, maintenance dosing of steroids appears to reduce the frequency and severity of relapse, as such a maintenance weaning regimen is proposed.

Conclusion: A practical, evidence-based flowchart and guidance recommendations with consensus from the EoE WG, education and research representatives of BSPGHAN was developed with detailed practical considerations for use in the UK.

Confidential: For Review Only

Key Messages

What is already known on this topic

- Therapeutic options for paediatric eosinophilic oesophagitis are limited. Swallowed topical steroids are the most successful treatment option in adults and children.
- Unlike in adults, currently there is no licensed formulation for a topical steroid in paediatric eosinophilic oesophagitis leaving prescribers with a variety of local dispensing policies.
- These therapies have evidence of benefit for paediatric patients of all ages but exact data on efficacy and safety have been missing in previous guidelines.

What this study adds

- Swallowed topical steroids probably provide improved remission rates for clinical symptoms and histology in children not responding to proton pump inhibitor (PPI) medication.
- In children < 10 years old, a standard total daily dose of 1mg/day can be safely used. In children ≥ 10 years old, a standard total daily dose of 2mg/day should be used.
- Expert consensus would favour a minimum of a 3-month induction period followed by repeat endoscopy with biopsies.
- Maintenance therapy should be considered for a minimum of 1-2 years. Usually, maintenance dosing is started at half the daily induction dose.
- In children, histological findings often correlate very poorly with symptoms.

How this study might affect research, practice or policy

- These recommendations and flowchart will help to standardise medical care of children with eosinophilic oesophagitis treated with swallowed topical steroids both nationally and internationally.
- Implementing a robust scheme for induction and maintenance with mandatory surveillance endoscopies will allow local benchmarking and prospective outcome measures.
- This study will close a gap of clinical research and provide a bridge for future studies on best treatment options and prognosis for children with eosinophilic oesophagitis.

Confidential: For Review Only

Introduction

Eosinophilic oesophagitis (EoE) is a chronic, immune or antigen-mediated oesophageal disease characterised by symptoms of oesophageal dysfunction and eosinophil predominant mucosal inflammation[1] first discovered in the early 1990s[2,3]. The rising prevalence is estimated at 15/100,000 before 2007 and 63/100,000 since 2017[4]. It is more common in males and is associated with atopic diseases. Current incidence estimates range from 5-10/100,000[5] similar to paediatric inflammatory bowel disease in the UK[6]. There has been a need to produce guidance for healthcare professionals and patients but these have mostly been adult focused and not always translatable to paediatric medicine. In the UK, a joint consensus guideline by the British Society of Gastroenterology and the British Society of Paediatric Gastroenterology, Hepatology and Nutrition (BSPGHAN) has attempted to redress this balance and pave the way for a joined up EoE approach[7].

Swallowed topical steroids (STS) have become common practice in recent years for induction and maintenance treatment of EoE, however practical barriers to treatment exist for the paediatric EoE (pEoE) cohort. The diagnosis is normally made in tertiary paediatric gastroenterology settings due to access to endoscopy and paediatric anaesthetists for general anaesthesia, these settings are less accessible for families living outside of major cities. Additionally, the newest orodispersible STS is unlicensed in patients less than 18 years old and is often unavailable in primary and secondary care. Tertiary centres are obliged to continue prescriptions which contributes to increased cost burden to families making long journeys to collect the medication or on hospital pharmacies who fund delivery of the medication to the family. Another barrier for pEoE is obtaining STS that are child-friendly and acceptable to the individual and family. Poor compliance is associated with medications that are difficult to administer due to product bitterness, time taken to prepare the medication and technical challenges involved in coordinating the swallow in younger age groups.

In the absence of a licensed paediatric formulation, choice of different STS and inequality of access to tertiary healthcare settings, UK practice varies and is not standardised. The BSPGHAN EoE working group (WG) has taken the opportunity to collaborate with BSPGHAN research leads and used Cochrane evidence to address some of the issues faced by children with EoE and provide healthcare professionals with practical, evidence-based guidance and a flowchart to support clinical care in healthcare settings.

Confidential: For Review Only

Methods

The BSPGHAN EoE WG met virtually by teleconference to discuss issues relevant to pEoE and what aspects guidance should focus on. There were four meetings between December 2021 and December 2022. The authors performed a systematic literature review on CENTRAL, MEDLINE, Embase, ClinicalTrials.gov and WHO ICTRP to 3 March 2023, with no prior date limits, using Cochrane guidance[8]. Search criteria were randomised controlled trials (RCTs) comparing any medical intervention or food elimination diet for the treatment of EoE. Dichotomous analysis was undertaken to assess response, either alone or in combination to any other intervention (including placebo). 2638 citations were identified and 18 RCTs pertaining to steroid use were included. This technical review and GRADE evidence included both adult and paediatric studies and informed the evidence base for the WG recommendations.

The authors and WG reviewed the following 7 themes in paediatric patients (age < 18 years) diagnosed with EoE.

1. What are the indications for STS?
2. Which STS preparations are best and how should they be made up?
3. Should dosing be based on age or height?
4. What dose and duration should induction therapy be?
5. What dose and duration should maintenance therapy be?
6. How and when should STS be reduced or stopped?
7. When should endoscopy be repeated?

Recommendations related to each theme were proposed and the WG voted anonymously on each recommendation. A 9-point scale was used with 9 representing strong agreement and 1 representing strong disagreement. Consensus was reached if more than 80% of eligible voting members voted 6, 7, 8 or 9. All WG members are professionals with expertise in EoE.

Patient and Public Involvement statement

Two members of the WG are affiliated with patient support groups; one is the chief executive officer of the EOSnetwork, an Eosinophilic Disease Charity that supports patients with EoE and their families, the other member is affiliated with GUTS UK, a charity funding research and supporting people living with gastrointestinal disease. Their involvement has been from the outset of development of this project with contribution and comment on the flowchart, statements and recommendations. They have been integral in considering how the information can be disseminated to the wider EoE community.

Confidential: For Review Only

Confidential: For Review Only

Results

All of the statements made by the BSPGHAN EoE WG (Table 1) for each pEoE theme reached consensus. Voting breakdowns are provided (Supplement 1).

Table 1: Recommendations from the BSPGHAN EoE WG with voting consensus percentage

Theme	The BSPGHAN EoE WG recommends that:	Voting consensus (%)
**1**	STS are indicated in:<br>• Induction therapy<br>• Maintenance therapy<br>• First line treatment of EoE<br>• Combination therapy with PPI<br>• Second line treatment after unsuccessful responses to dietary therapy and/or PPI<br>• Oesophageal stricture treatment in isolation or as an adjunct to oesophageal balloon dilatation	100<br>100<br>92<br>83<br>100<br>92
**2**	• The type of STS preparation offered should take into consideration the child's age, palatability, chance of adherence, comorbidities and family support<br>• If a child is assessed to be able to tolerate and coordinate orodispersible budesonide (tablet) preparation and it is locally available, then this preparation should be first choice	100<br>92
**3**	• Age should be used to determine STS dosing unless a child significantly deviates from their growth centiles, in which case height should be accounted for	100
**4**	• Twice daily dosing is considered for induction therapy<br>• For children < 10 years old, a dose of 0.5mg twice per day should be used (1mg/day), for children ≥ 10 years old, a dose of 1mg twice per day should be used (2mg/day)<br>• Induction therapy should usually be given for a minimum of 3 months	100<br>100<br>100
**5**	• After histological remission has been achieved, maintenance therapy should be considered for a minimum of 1-2 years<br>• For children < 10 years old, a dose of 0.5mg/day should be used, for children ≥ 10 years old, a dose of 1mg/day should be used	100<br>100
**6**	• After confirmed histological remission and 3-month induction therapy, clinicians should consider halving the STS dose	100<br>92

	• During maintenance therapy, dose weaning should be considered every 6-12 months<br>• Oral or oesophageal candidiasis does not usually require stopping the STS; antifungal treatment should be added alongside the STS	83
**7**	Endoscopy:<br>• Should be repeated during the induction period to ensure histological response to STS and allow weaning of the medication<br>• Should be repeated if there is worsening of symptoms / oesophageal dysfunction<br>• Surveillance should be considered at 1-2 yearly intervals or if considering stopping treatment or following cessation of therapy if clinically indicated	92<br><br>100<br><br>100

Theme 1 - What are the indications for STS?

There is strong evidence for the use of STS in EoE. STS have very good remission rates, 71% symptomatically and 59% histologically in children not responding to proton pump inhibitor (PPI) medication[8,9]. There is emerging evidence showing that combination therapy of STS with PPI may lead to even better outcomes[8]. Currently, there are no data in pEoE showing that the use of STS will prevent oesophageal strictures or that strictures can be treated in isolation with STS. However, in practice, medical treatment is often trialled before therapeutic balloon dilatation. The main evidence supporting steroid use in moderate to severe oesophageal strictures is by using systemic steroids. A multicentre retrospective cohort study found that with systemic steroids 20/20 patients showed clinical improvement, 15/20 became asymptomatic and 19/20 had stricture resolution at endoscopy[10].

Theme 2 - Which STS preparations are best and how should they be made up?

There are three main STS preparations: a) oral viscous budesonide ('budesonide slurry') formed from budesonide respules combined with a viscous binding agent, b) orodispersible budesonide (tablets) which melt on the tongue before being swallowed, c) inhaled fluticasone sprayed into the buccal cavity and swallowed. There are currently no head-to-head trials on different preparations or on the ideal viscous binding agent to use to form the slurry. Expert opinion would advocate using an age appropriate preparation which is generally a budesonide slurry in younger children and orodispersible tablets in older children.

Theme 3 - Should dosing be based on age or height?

Research studies looking at histological remission rates and dosing in children with EoE have used either age ranges (< 10 years or ≥ 10 years) or heights (< 1.5metres or ≥ 1.5metres) to define cut offs for medication dosing[11]. Expert opinion would advocate using age for simplicity. If a child deviates from their expected growth centiles then consideration should be given to using height instead of age to determine STS dosing.

Theme 4 - What dose and duration should induction therapy be?

Confidential: For Review Only

There is a lack of evidence surrounding the duration and dosing regimen of induction therapy. Expert consensus would favour a minimum of a 3-month induction period and twice daily dosing to promote increased contact time between the medication and oesophageal mucosa (most clinical studies have been conducted with twitch twice daily dosing).

In adults, data from randomised placebo-controlled trials show that in a dosage of 1mg twice daily, orodispersible budesonide achieved remission after 12 weeks in 85% of patients[12]. Viscous budesonide formulations using sucralose or other substrates (evaluated in a prospective randomised placebo-controlled trial only) may be equally, less or more effective.

In children < 10 years old, a standard total daily dose of 1mg/day in divided doses can be safely used. In children ≥ 10 years old, a standard total daily dose of 2mg/day in divided doses should be used. In select cases doses of up to 2.8mg/day (<10 years old) and 4mg/day (≥10 years old) have been used[13]. Clinician judgement should be used to balance up the optimal dosing frequency versus the chance of adherence in particular cases.

Theme 5 - What dose and duration should maintenance therapy be?

Once histological remission has been accomplished, use of maintenance dosing of steroids appears to reduce the frequency and severity of relapse[9,14]. There is currently limited evidence regarding how long maintenance therapy should be continued for. In adults, data from randomised placebo-controlled trials show that a dosage of orodispersible budesonide of 0.5mg or 1mg twice daily achieved persistent remission after 48 weeks in 73.5% and 75%, respectively[14]. The viscous formulations using sucralose or other substrates (evaluated in a prospective randomised placebo-controlled trial only) may be equally, better or less effective.

Once histological remission has been achieved during the induction phase, maintenance therapy should be considered for a minimum of 1-2 years. Usually, maintenance dosing is started at half the daily induction dose. In the majority of published clinical studies, STS are given twice daily (18 studies) and only in 3 studies once daily (evening) and 1 study four times daily[9]. Outside of clinical studies, in reality, as advised by our corresponding patient support organisations, patients and families sometimes struggle with the logistics of more frequent (more than once daily) dosing with the morning dose often missed[15]. Therefore, we recommend that consideration should be given and discussion take place to whether once or twice daily dosing is likely to result in improved adherence to longer term therapy and achievement of the desired effect. There is no data suggesting that twice daily dosing determines an increased risk and no data indicating that once daily dosing has the same treatment effect. From a practical perspective, principles of dental hygiene and exposure of the oesophagus to the drug for over 30 minutes with no food or drink allowed, determine which logistics are feasible for children and families on a daily basis.

Theme 6 - How and when should STS be reduced or stopped?

Histological remission is defined as < 15 eosinophils per high power field or 0.3mm^2 in any oesophageal biopsy examined (proximal, mid and distal oesophagus). Systemic side effects of topical steroids are considered rare during the long-term treatment of EoE patients in those naïve to STS and without pre-existing adrenal conditions[16]. However EoE patients often have associated atopic conditions and careful consideration should be given to the total daily steroid dose and steroid burden that these patients receive especially during seasonal exacerbations of their atopic disease. Consideration should be given to maintaining EoE on the lowest practical dose of STS to maintain histological remission.

Confidential: For Review Only

In a systematic review on adrenal insufficiency in children with EoE, adrenal testing was abnormal in 15.8% , however adrenal insufficiency between topical steroids and placebo was not statistically different over 2-12 weeks of treatment[17]. In observational studies, the risk of adrenal insufficiency increased from 0-10% up to 30-66% in patients with multiple steroid treatments (e.g. for asthma or rhinitis)[17]. In a multicentre study from the USA, 5% of children on STS were considered to show signs of adrenal gland insufficiency while on STS for longer than 6 months[16].

Children with symptoms suggestive of adrenal insufficiency or risk factors for developing the condition (e.g. patients receiving multiple steroids or on twice daily induction regimens for more than 6 months) should be evaluated as per local protocols. The short synacthen test (low dose injected adrenocorticotropic hormone) is the preferred investigation in most centres[17].

Continued monitoring of growth, bone mineral density and adrenal suppression is recommended in children and adolescents[7]. Subtle decrease in linear growth was observed in a retrospective multicentre study in children with EoE receiving topical steroid treatment and sex differences were observed affecting girls treated with combined elemental diet and topical steroids[18]. Children with nutritional deficits (e.g. secondary to behavioural or dietetic restrictions) should be carefully monitored for adequate growth (e.g. centile, nutritional assessment, biochemically) and bone health (e.g. DEXA scan)[19].

Theme 7 - When should endoscopy be repeated?

There is no clear paediatric guidance on when an endoscopy should be repeated following diagnosis. In children, histological findings often correlate very poorly with symptoms[9,20]. Endoscopy and biopsy should be used to ensure that STS treatment strategies are effective, enable management changes and investigate worrying symptoms.

Flowchart

By assimilating the key evidence from the literature and Cochrane review (Figure 1), recommendations (Table 1) and expert consensus, a practical easy to use flowchart (Figure 2) was designed to help support healthcare professionals involved in the management of pEoE.

Confidential: For Review Only

Confidential: For Review Only

Discussion

The incidence, prevalence, recognition and management of EoE has rapidly changed over the past 30 years and will continue to evolve as our understanding and therapeutic options change. It is essential that guidance and recommendations are standardised to ensure patients are not disadvantaged by variations in access to healthcare professionals, specific professional knowledge or medications. Using the latest Cochrane systematic review and through collaboration between a national EoE expert group, BSPGHAN education and research leads and patient support groups, we recommend guidance that addresses common themes in pEoE in the UK. We have produced a quick reference flowchart that can be used at the bedside to help guide professionals involved in the management of pEoE. The flowchart was orally presented at the 2023 BSPGHAN Annual Meeting attended by 130 professionals for wider society member review and discussion. Specific comments were requested using a QR code to ensure stakeholder engagement before wider dissemination. The flowchart was found to be useful by all responders with respondents keen to use it in their practice. Where evidence exists, this has been used to strengthen the recommendations and flowchart and where lacking, expert consensus has been followed.

Efficacy, palatability and adherence

Twice daily dosing theoretically allows for a minimum of a daily anti-inflammatory dose especially if one dose is missed. The WG were advised that for adherence to maintenance therapy once daily dosing is more acceptable[15]. Individual circumstances should be discussed and tailored according to lifestyle and activities. As there are currently no licensed preparations of STS in children, a variety of carrier substances for budesonide STS preparations to coat the oesophagus have been used for administration and palatability (Appendix 1[21-26]). This is heavily reliant on local expertise and there are no head to head trials to advocate use of one over another. Orodispersible budesonide (tablets) are licenced in adults and are increasingly being used off label. Prospective paediatric studies for STS have been conducted and first results have been submitted for peer-reviewed presentation[27], indicating good prospects for suspensions and tablets, subject to pharmaceutical licensing, for future application in children.

Side effects

Children with EoE have a 70% association with atopic comorbidity, steroids are often prescribed by other physicians (Dermatology, Respiratory, General Paediatrics) which potentially increases the cumulative steroid burden. The largest multicentre study reported that 5% of children showed signs of adrenal gland insufficiency while on STS for EoE for longer than 6 months[16]. In study protocols, baseline cortisol levels, short synacthen testing and follow-up while on maintenance treatment have been applied, however there is controversy of the standardisation and interpretation of test results, we recommend to perform these as clinically indicated and according to local endocrinology advice. Other monitoring should include growth parameters and bone mineral densities. Candida infection is rare and can be treated simultaneously with topical antifungal or oral medication[7].

Endoscopy, prognosis, withdrawal and escalation

In the absence of validated clinical activity scores or non-invasive biomarkers, the group agree that endoscopic reassessment with histology should be used after any significant treatment start or modification. As STS are effective in more than 80% of children when taken according to protocols and usually well tolerated, prognosis is very good. Withdrawal is indicated in non-responders, disease

progression under therapeutic dosage of STS or clinically relevant adrenal gland insufficiency. We found limited evidence for escalation of treatment with regard to dosage of STS[18,20].

Limitations

There is no evidence for the ideal carrier substance used to generate the optimal formulation of STS. There is a lack of studies to compare potential effects of once or twice daily administration of a defined daily dose of STS on efficacy and long-term outcomes. STS have been considered mainly in isolation and combination therapy has not been investigated as they have been discussed elsewhere[8,18,20,28].

Implications for clinicians and policymakers

This project is a starting point for pEoE in the UK and beyond; further work should be considered with other professionals in paediatric practice to develop a collaborative multi-system approach to pEoE care including allergy and transition to adult settings. This would ensure continued evolution of the clinical reference flowchart when better access to medication occurs; merging paediatric and adult practice should be the gold standard to aim for.

Unanswered questions and future research

Increasing the pEoE knowledge base is vital to combat the increasing disease burden. Differentiating if the increased pEoE prevalence is due to earlier recognition or is suggestive of a more severe phenotype may help to risk stratify patients and impact ongoing surveillance and transition decisions. Whilst it is important to research new treatments for EoE and improve access to these in paediatrics, it will be crucial to know whether the stimulation and recruitment of eosinophils into the oesophagus can be prevented. More trials are needed on whether the type, viscosity and volume of the carrier solution affects the efficacy of topical budesonide. Using this guidance as a baseline will allow future research into best practice and prognosis of pEoE.

Conclusion

Evidence-based guidance and a flowchart with consensus from the EoE Working Group and education and research representatives of BSPGHAN was developed with detailed practical considerations for use in the UK and beyond.

Research Ethics Approval

No ethics approval needed

Funding statement

This research received no specific grant from any funding agency in the public, commercial or not-for-profit sectors

Confidential: For Review Only

Competing Interests statement

Mixed competing interests: All authors have completed the ICMJE uniform disclosure form at http://www.icmje.org/disclosure-of-interest/ and declare: no support from any organisation for the submitted work; MKHA has received research grants and honorariums from BSPGHAN, Guts UK, Dr Falk Pharma, Mead Johnson, Abbott and Nutricia; AC has received grant funding from AZ, Bristol Myers Squibb, AVIR, Allakos, Dr Falk Pharma and Sanofi / Regeneron and advisory panel honorarium was received from AZ, Sanofi / Regeneron, AVIR and Dr Falk Pharma; Guts UK Charity do receive funding for the Falk Awards from Dr Falk Pharma; no other relationships or activities that could appear to have influenced the submitted work.

Contributor and guarantor information:

Joseph Chan and Diana Flynn had the idea for this project on behalf of the BSPGHAN EoE Working group. The initial paper search and outline of the article were developed by them. Joseph Chan took the lead on writing the paper. Diana Flynn contributed to the writing of the paper and development of the steroid algorithm.

Morris Gordon performed the detailed Cochrane Review whereby corroborating evidence was obtained and contributed to structure and writing of the paper.

Marco Gasparetto contributed to the writing of the paper, and advice on grading of statements and recommendations.

Marcus KH Auth took senior overview of the development of the paper and algorithm as Chair of the BSPGHAN EoE Working Group, and contributed to writing and editing of the paper.

All members of the BSPGHAN EoE working group contributed to review of the steroid algorithm, voting and corroboration of statements.

Guarantor for the accuracy and clinical governance of the manuscript is Marcus KH Auth as chair of the EoE working group and member of ESPGHAN EGID working group.

The corresponding author attests that all listed authors meet authorship criteria and that no others meeting the criteria have been omitted.

The lead author affirms that the manuscript is an honest, accurate, and transparent account of the study being reported, and that no important aspects of the study have been omitted. Any discrepancies from the study as originally planned have been explained.

Copyright/licence for publication

The Corresponding Author has the right to grant on behalf of all authors and does grant on behalf of all authors, a worldwide licence to the Publishers and its licensees in perpetuity, in all forms, formats and media (whether known now or created in the future), to i) publish, reproduce, distribute, display and store the Contribution, ii) translate the Contribution into other languages, create adaptations, reprints, include within collections and create summaries, extracts and/or, abstracts of the Contribution, iii) create any other derivative work(s) based on the Contribution, iv) to exploit all subsidiary rights in the Contribution, v) the inclusion of electronic links from the Contribution to third

Confidential: For Review Only

party material where-ever it may be located; and, vi) licence any third party to do any or all of the above.

Figure legends

Figure 1. Summary of cochrane evidence

Figure 2. Management flow chart / algorithm

References

1 Furuta GT, Katzka DA. Eosinophilic Esophagitis. *N Engl J Med.* 2015;373:1640-8.

2 Attwood SE, Smyrk TC, Demeester TR, *et al.* Esophageal eosinophilia with dysphagia. A distinct clinicopathologic syndrome. *Dig Dis Sci.* 1993;38:109-16.

3 Straumann A, Spichtin HP, Bernoulli R. [Idiopathic eosinophilic esophagitis: a frequently overlooked disease with typical clinical aspects and discrete endoscopic findings]. *Schweiz Med Wochenschr.* 1994;124:1419-29.

4 Navarro P, Arias Angel, Arias-Gonzalez L, *et al.* Systematic review with meta-analysis: the growing incidence and prevalence of eosinophilic oesophagitis in children and adults in population-based studies. *Aliment Pharmacol Ther.* 2019;49:1116-25.

5 Dellon ES, Hirano I. Epidemiology and natural history of eosinophilic esophagitis. *Gastroenterology.* 2018;154:319-32.

6 Henderson P, Rogers P, Gillett P, et al. The epidemiology and natural history of paediatric inflammatory bowel disease in a UK region: a prospective 14-year study. *Arch Dis Child.* 2012; 97: A53-A54.

7 Dhar A, Haboubi H, Auth M, Attwood S. Eosinophilic oesophagitis: improving diagnosis and therapy – reducing the burden of repeated endoscopy. *Frontline Gastroenterol.* 2022:13(e1):e51-e56. doi: 10.1136/flgastro-2022-102118. eCollection 2022.

8 Franciosi JP, Gordon M, Sinopoulou V, Dellon ES, Gupta SK, Reed CR, et al. Medical treatment of eosinophilic esophagitis. *Cochrane Database Syst Rev* 2023; 2023(7): CD004065. https://www.cochranelibrary.com/cdsr/doi/10.1002/14651858.CD004065.pub4/information

9 Oliva S, Dias JA, Rea F, et al. Characterization of Eosinophilic Esophagitis from the European Pediatric Eosinophilic Esophagitis Registry (pEEr) of ESPGHAN. *J Pediatr Gastr Nut*r. 2022;75(3):325-333.

10 Hoofien A, Rea F, ESPGHAN EGID Working Group et al. Systemic steroids have a role in treating esophageal strictures in paediatric eosinophilic esophagitis. *Dig Liver Dis.* 2021;53(3):324-328.

11 Gupta SK, Hill M, Vitanza JM, Farber RH, Desai NK, et al. Pharmacokinetics of Budesonide Oral Suspension in Children and Adolescents with Eosinophilic Esophagitis. *J Pediatr Gastroenterol Nutr.* 2022 Aug 1;75(2):186-191. doi: 10.1097/MPG.0000000000003482. Epub 2022 Jun 6.PMID: 35666852

12. Lucendo AJ, Miehlke S, Schlag C, et al. Efficacy of Budesonide orodispersible tablets as induction therapy for eosinophilic esophagitis in a randomised, placebo-controlled trial. *Gastroenterology* 2019; 157(1): 74-86.e15.

13 Gupta SK, Collins MH, Lewis JD, et al. Efficacy and safety of oral budesonide suspension (OBS) in pediatric subjects with eosinophilic esophagitis (EoE): results from the double-blind, placebo-controlled PEER study. *Gastroenterology.* 2011;140:S179.

14 Straumann A, Lucendo AJ, Miehlke S, et al. Budesonide orodispersible tablets maintain remission in a randomised, placebo-controlled trial of patients with eosinophilic esophagitis. *Gastroenterology.* 2020; 159(5): 1672-85 e5.

15 Auth MKH, Chan J, Flynn D, personal communication with A Cordell (Eosnetwork) and J Thompson (Guts UK!), 09-Mar-2023.

16 Bose P, Kumar S, Nebesio TD, Li C, Hon EC, et al. Adrenal Insufficiency in Children With Eosinophilic Esophagitis Treated With Topical Corticosteroids. *J Pediatr Gastroenterol Nutr.* 2020, 70(3):324-329.

17 Philpott H, Dougherty MK, Reed CC, Caldwell M, Kirk D, et al. Systematic review: adrenal insufficiency secondary to swallowed topical corticosteroids in eosinophilic oesophagitis. *Aliment Pharmacol Ther*. 2018 Apr;47(8):1071-1078.

18 Oliva S, Amil Dias J, Rea F, Malamisura M, Espinheira MC, et al. Characterization of Eosinophilic Esophagitis in a Multinational Prospective Pediatric Cohort: Initial Report from the European Pediatric Eosinophilic Esophagitis Registry (pEEr) of ESPGHAN. *J Pediatr Gastroenterol Nutr.* 2022; 75(3): 325-333.

19. Jensen ET, Huang KZ, Chen HX, Landes LE, McConnell CA, et al. Longitudinal Growth Outcomes Following First-line Treatment for Pediatric Patients With Eosinophilic Esophagitis. *J Pediatr Gastroenterol Nutr.* 2019; 68(1): 50-55.

20 Hoofien A, Dias JA, Malamisura M, Rea F, Chong S, et al. Pediatric Eosinophilic Esophagitis: Results of the Retrospective Pediatric Eosinophilic Esophagitis Registry (RetroPEER). *J Pediatr Gastroenterolg Nutr.* 2019 Apr;68(4):552-558.

21 Syverson EP, Tobin M, Patton T, Franciosi JP, Gupta SK, et al. Variability in Swallowed Topical Corticosteroid Practice Patterns for Treatment of Pediatric Eosinophilic Esophagitis. J Pediatr Gastroenterol Nutr 2023 Aug 1;77(2):256-259. doi: 10.1097/MPG.0000000000003834. Epub 2023 May 19.

22 Mukkada VA, Gupta SK, Gold BD, Dellon ES, Collins MH, et al. Pooled Phase 2 and 3 Efficacy and Safety Data on Budesonide Oral Suspension in Adolescents with Eosinophilic Esophagitis. J Pediatr Gastroenterol Nutr. 2023 Sep 18. doi: 10.1097/MPG.0000000000003948. Online ahead of print.PMID: 37718471.

23 Debras C, Chazelas E, Srour B, Druesne-Pecollo N, Esseddik Y, et al. Artificial sweeteners and cancer risk: Results from the NutriNet-Santé population-based cohort stud. PLoS Med. 2022 Mar; 19(3): e1003950. Published online 2022 Mar 24. doi: 10.1371/journal.pmed.1003950.. Debras et al.

24 Debras C, Deschasaux-Tanguy M, Chazelas E, Sellem L, Druesne-Pecollo N, Esseddik Y Artificial Sweeteners and Risk of Type 2 Diabetes in the Prospective NutriNet-Santé Cohort.Diabetes Care. 2023 Sep 1;46(9):1681-1690. doi: 10.2337/dc23-0206

Confidential: For Review

25 Debras C, Chazelas E, Sellem L, Porcher R, Druesne-Pecollo N. et al. Artificial sweeteners and risk of cardiovascular diseases: results from the prospective NutriNet-Santé cohort. BMJ. 2022 Sep 7;378:e071204. doi: 10.1136/bmj-2022-071204.

26 Mukkada VA, Gupta SK, Gold BD, Dellon ES, Collins MH, Katzka DA, Falk GW, Zhang W, Boules M, Hirano I, Desai N. Pooled Phse 2 and 3 Efficacy and Safety Data on Budesonide Oral Suspension in Adolescents with Eosinophilic Oesophagitis. J Pediatr Gastroenterol Nutr. 2023 Sep 18;77(6):760-768. doi: 10.1097/MPG.0000000000003948. Online ahead of print.

27 Lucendo AJ, Antunes H, Papadopoulou A, Auth MKH, Dutt S, et al. Budesonide oral suspension is effective and safe in pediatric eosinophilic esophagitis (EoE): results from the induction phase of the randomised, double-blind, placebo-controlled trial PEDEOS-1. Abstracts submitted to UEGW and ESPGHAN 2024, 13-dec-2023.

28 Amil Dias J, Salvatore S, Papadoloulou A, Thomson M, Gutierrez-Junquez C, et al. Updated Guidelines for Diagnosis and Management of Eosinophilic Esophagitis in Pediatrics. Position Paper for Diagnosis and Management by the EGID Working Group of ESPGHAN. *J Pediatric Gastroenterol Nutr 2024, in print.*

Confidential: For Review Only
